# Coronary heart disease and type 2 diabetes metabolomic signatures in the Middle East

**DOI:** 10.3389/fendo.2025.1531525

**Published:** 2025-11-17

**Authors:** Mohamed Elshrif, Keivin Isufaj, Ayman El-Menyar, Ehsan Ullah, Alka Beotra, Mohammed Al-Maadheed, Vidya Mohamed-Ali, Mohamad Saad, Jassim Al Suwaidi

**Affiliations:** 1 Qatar Computing Research Institute, Hamad Bin Khalifa University, Doha, Qatar; 2 Clinical Research, Trauma & Vascular Surgery, Hamad Medical Corporation, Doha, Qatar; 3 Department of Clinical Medicine, Weill Cornell Medical College, Doha, Qatar; 4 Anti-doping Lab Qatar, Doha, Qatar; 5 Department of Cardiology, Heart Hospital, Hamad Medical Corporation, Doha, Qatar

**Keywords:** type 2 diabetes, coronary heart disease, metabolomics, Middle Eastern populations, supervised learning, predictive modeling, pathway enrichment analysis, metabolite risk score

## Abstract

**Background:**

The growing field of metabolomics has opened new venues for identifying biomarkers of type 2 diabetes (T2D) and predicting its consequences, such as coronary heart disease (CHD). Despite their large size, Middle Eastern populations are underrepresented in omics research. In this study, we aim at investigating metabolomics profiles of T2D stratified by the CHD comorbidity for Middle Eastern population, such as Qatari population.

**Methods:**

In this cross-sectional study, we used a total of 641 metabolites from a large cohort of 3,679 Qatari adults from the Qatar BioBank (QBB; 272 T2D and 2,438 non-T2D individuals) and Qatar Cardiovascular Biorepository (QCBio; all CHD patients; 488 T2D and 481 non-T2D individuals). Univariate and pathway enrichment analyses were performed to identify metabolites associated with T2D in the absence or presence of CHD. Machine learning (ML) models, and metabolite risk scores were developed to assess the predictive power of the different combinations of T2D and CHD.

**Results:**

Many metabolites were significantly associated with T2D in both the QBB and QCBio cohorts. Among these, we observed 1,5-anhydroglucitol (1,5-AG) (P = 1.33 × 10^−68^ [-5.20, -4.16] in QBB vs 9.82 × 10^−33^ [-2.51, -1.80] in QCBio), glucose (P = 7.14 ×10^−57^ [4.09, 5.23] in QBB vs. 3.26 × 10^−29^ [1.41, 2.00] in QCBio), and mannose (P = 2.61 × 10^−54^ [2.68, 3.45] in QBB vs. 1.01 × 10^−27^ [1.45, 2.09] in QCBio). Other metabolites were significantly associated with T2D only in one cohort, e.g., gamma-glutamylglutamine (P = 1.79 × 10^−20^ and *β* = -2.61 in QBB vs. P = 5.12 × 10^−1^ and *β* = 0.10 in QCBio). The enriched pathways (FDR P< 0.05), common to both cohorts, included galactose metabolism and valine leucine, and isoleucine biosynthesis and degradation. Few pathways were significantly associated with T2D in only one cohort: fructose and mannose, and Pantothenate and CoA biosynthesis metabolisms were significant in the QCBio cohort, whereas Arginine biosynthesis, and Alanine, aspartate and glutamate metabolisms were significant in the QBB cohort. ML models performed well in predicting T2D with high accuracy (*>*80% in both QBB and QCBio). The metabolite risk score (MRS) developed in the QCBio and tested in the QBB while adjusting for hemoglobin A1C yielded an odds ratio (OR) of 21.18 for the top quintile vs. the remaining quintiles.

**Conclusions:**

Metabolomic profiling has the potential for the early detection of metabolic alterations that precede clinical symptoms of T2D and CHD in the presence of T2D. Risk scores showed great performance in predicting T2D and CHD, but longitudinal data are required to provide evidence for disease risk. Early detection allows timely interventions and improved management strategies for both T2D and CHD patients.

## 1 Introduction

The number of people, who live with diabetes is quickly increasing globally, driven by many factors including ageing, urbanization, and the growing prevalence of obesity (1–3). Diabetes is estimated to affect more than 500 million people worldwide, with severe impacts on health and the economy (4). The prevalence of diabetes is continuously increasing worldwide, and it is expected that the number of patients with diabetes will approach 550 million by 2030 and 700 million by 2045 (5–7). However, the burden of Type 2 Diabetes (T2D) is not shared equally between different ethnic groups globally. Non-White ethnic populations are three-to-five times higher prevalence of T2D than people of White-European background (8). In the US several recent studies discussed disparities in the prevalence of diabetes (9–12). For example, Cheng et al. (10), showed that the Hispanic American adults having the highest prevalence with 22.1% followed by non-Hispanic Black with 20.4%, and non-Hispanic Asian American with 19.1% compared with the non-Hispanic White American adult population with 12.1%. South Asians ethnic populations develop T2D five-to-ten years earlier and possess higher risk of developing T2D compared to White European population (two-to six-fold) (13). Similarly, 9% of White European population are diagnosed with T2D under the age of 40 years compared with the Black African-Caribbean populations of 23% (14). The Middle East, North Africa, and especially the Gulf region show a high prevalence of diabetes, exceeding 17% in some countries, such as Qatar (4, 15). In addition, the incidence of young-onset diabetes is rapidly increasing in Gulf countries (all countries that have coasts in the Arabic Gulf, which includes Kuwait, Saudi Arabia, Bahrain, Qatar, Emirates, and Oman). These populations have high rates of metabolic syndrome at a young age, with a prevalence 10-15% higher than that in most developed countries (16). The etiology of type 2 diabetes (T2D) is complex and is associated with diverse complications (17–20). Individuals with T2D have greater cardiovascular morbidity and mortality, and the risk of cardiovascular disease (CVD) and CVD-related death is two to four times greater among T2D patients (21–23). A recent study showed that two-thirds of deaths in patients with T2D are caused by CVD (24). Furthermore, the risk of developing coronary heart disease (CHD) and heart failure (HF) in T2D patients increased two-to-four fold and two-to-eight fold, respectively (25–27). Prevention and early detection of T2D are crucial for improving treatment to avoid/delay major complications (28–31). T2D and CHD are partly caused by complex interactions between genetic and metabolic profiles (32–34). Metabolic alteration is the leading hallmark of diabetes, as it was assumed that T2D individuals’ metabolic pathways are affected and play an important role in their total metabolomic dysfunction (35, 36). For example, in one study Chen et al. (36), impaired glucose metabolic homeostasis led to hyperglycemia, which is a hallmark of diabetes mellitus. Numerous studies have investigated the associations between metabolites and T2D (30, 37, 38) and between metabolites and CHD (39–41). Stratification of metabolomics signatures of T2D patients with respect to CHD is important to shed light on the biological mechanisms of these two diseases. The relationship at the metabolomics level for T2D and CHD was studied previously (42), but to the best of our knowledge, this has rarely been examined for Middle Eastern populations. Hence, this is the first large-scale metabolomics study of T2D and CHD in a Qatari population, providing insights from an underrepresented region. The identification of differential metabolic profiles of T2D patients who have CHD vs. T2D patients who do not develop CHD may lead to therapeutic approaches to reduce the occurrence of CHD in T2D patients. In this study, we assessed the differences between the metabolomics profiles of T2D patients stratified by the absence or presence of CHD in a Middle Eastern (Qatari) dataset using univariate and multivariate analyses, pathways enrichment analysis, machine learning, and metabolite risk score analysis. Metabolomic data were generated by Metabolon for two cohorts collected by the Qatar BioBank [QBB, 2,710 samples, (43)] and Qatar Cardiovascular Biorepository (QCBio, 969 samples, (40, 44, 45).

## 2 Materials and methods

### 2.1 Study cohort

Our cross-sectional study included two cohorts: (1) the QBB cohort, which comprised 2,710 participants (272 T2D patients and 2,438 non-T2D controls), none with CHD based on the survey completed by participants, and (2) the QCBio cohort, which comprised 969 CHD patients (481 T2D patients and 488 non-T2D patients) (**Supplementary Figure 1**). CHD patients were identified from the Cardiac Catheterization Laboratory, Coronary Care Unit, and Heart Hospital Clinics at Hamad Medical Corporation (HMC), Doha, Qatar. Patients with a history of acute coronary syndrome or stable angina were included in the study (44). For patients with CHD and T2D, T2D occurs first. The study was approved by the Institutional Review Boards of HMC and QBB. Written informed consent was obtained from all patients before their participation. The cohorts’ characteristics are shown in **Table 1**. The diagnostic criteria used for identifying T2D and CHD were as follows: T2D status in QCBio cohort was defined as fasting blood glucose ≥ 126 mg/dL, random glucose ≥ 200 mg/dL, hemoglobin A1C ≥ 6.5%, or a prior diagnosis with oral hypoglycemic or insulin therapy. Within QBB cohort, patients with hemoglobin A1C ≥ 6.5% were considered to have T2D. The age at onset of both T2D and CHD was not available in our dataset, which prevented the analysis accounting for age at onset.

**Table 1:** Cohorts characteristics.

Cohort Name	Diabetes Status	Gender	Participants N (%)	Age, Mean (SD) years	P value (Gender ∼ Age)	BMI, Mean (SD) *kg.m*^−2^	P value (Gender ∼ BMI)	Hypertension
QCBio	T2D+	Females Males All	179 (37.21) 302 (62.79) 481 (100)	59.07 (10.05) 58.96 (11.29) 59.01 (10.84)	9.13E-01	34.08 (6.38) 30.05 (5.11) 31.55 (5.93)	1.63E-11	148 243 391
	T2D-	Females Males	191 (39.14) 297 (60.86)	44.57 (15.21) 47.64 (15.09)	2.96E-02	29.56 (5.04) 29.51 (4.86)	9.12E-01	41 (10 NA) 100 (12 NA)
		All	488 (100)	46.45 (15.20)		29.53 (4.93)		141 (22 NA)
QBB	T2D+	Females Males All	137 (50.37) 135 (49.63) 272 (100)	53.59 (8.98) 48.81 (10.84) 51.22 (10.20)	1.66E-04	33.24 (6.24) 29.93 (5.52) 31.60 (6.12)	2.05E-05	NA
	T2D-	Females Males	1191 (48.85) 1247 (51.15)	38.34 (11.88) 37.92 (10.65)	3.51E-01	28.82 (6.20) 28.40 (5.40)	7.43E-02	
		All	2438 (100)	38.13 (11.57)		28.60 (5.81)		

T2D status in the QCBio dataset was defined as fasting blood glucose ≥126 mg/dL, random glucose ≥ 200 mg/dL, hemoglobin A1C ≥ 6.5%, or a prior diagnosis with oral hypoglycemic or insulin therapy. Within controls, hemoglobin A1C ≥ 6.5% was used to define T2D patients. T2D+: T2D patient; T2D-: non-T2D patient. * is part of the metabolite name↑ increased, ↓decreased.

As indicated in **Table 1**, for QCBio cohort, the number of T2D patients was almost matched with non-T2D individuals and the females represent 38%, whereas the males represent 62%. For T2D patients, the age and BMI were statistically different between males vs females with P = 0.91 and P = 1.63 × 10^−11^, respectively. The number of hypertension individuals was 391 patients. For non-T2D individuals, the age and BMI were statistically different between males vs females with P = 0.03 and P = 0.91, respectively. The number of hypertension individuals was 141 patients. For QBB cohort, the number of T2D patients was 272, whereas the non-T2D individuals was 2,438. The gender distribution was equal between females and males. The age and BMI were statistically different between T2D for females vs males with P = 1.66 × 10^−4^ and P = 2.05 × 10^−5^, respectively, whereas P = 0.35 and P = 0.07, respectively for non-T2D individuals. None of the QBB cohort individuals has hypertension.

### 2.2 Metabolomics profiling and data quality control

**Figure 1:**
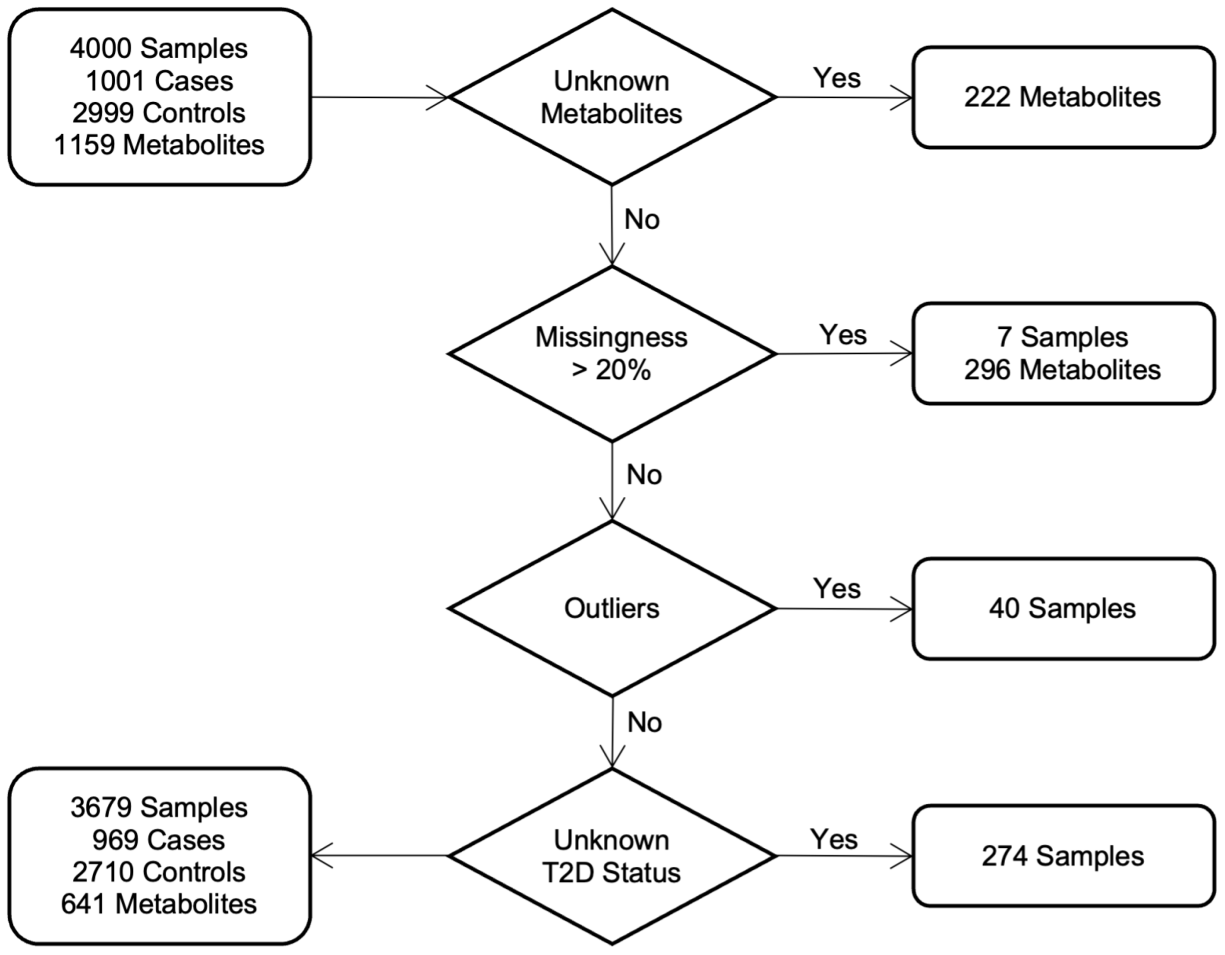
Study cohorts and quality control: workflow diagram indicates the selection process of individuals in both cohorts.

Serum metabolites for the QCBio and QBB cohorts were jointly quantified by untargeted, ultrahigh-performance liquid chromatography-tandem mass spectrometry (UPLC-MS/MS) and curated by Metabolon Inc. We used the HD4 platform, which exactly mimics and is accredited by Metabolon Inc. More specifically, we used 96-well plates, with 40 samples per plate, each with 5 internal QCs, 3 blanks and 1 pooled sample prepared from the 144 samples, to check for any drift between plates (46, 47). The obtained data were normalized across batches to generate batch-normalized data and to correct for minor instrument technical variation that could occur from one batch to another. Each compound was corrected in instrument batch blocks by registering the medians of each batch to equal one and normalizing each data point proportionally. A total of 641 out of the 1,159 metabolites were analyzed from both cohorts in our study after standard quality control steps were performed by Ullah and his colleagues (40). **Figure 1** shows the details of the selection process in both cohorts. Briefly, 296 metabolites and seven samples with *>* 20% missing data were discarded. Principal component analysis (PCA) was used to detect and remove 40 outliers from our dataset, with a criterion based on the first five principal component values falling outside the range of [*µ* ± 5 SD]. To mitigate the influence of extreme values in the metabolite data, metabolites were winsorized using 80% winsorization: values for a metabolite below the 10*^th^* percentile were set to the 10*^th^* percentile, and values above the 90*^th^* percentile were set to the 10*^th^* percentile. For more details, see **Supplementary Material**.

### 2.3 Statistical analysis

Statistical and machine learning analyses were performed with R software (version 4.1.2) and Python software (version 3.11.13) for both cohorts. The packages’ versions are detailed in Supplemental Material.

#### 2.3.1 Univariate analysis

The first analysis was conducted to compare T2D vs non-T2D patients in the QCBio cohort, where 136 all individuals had CHD. The same analysis was performed in the QBB cohort as a replication stage for the QCBio results. The analysis was separately performed in each cohort using logistic regression, adjusting for age, sex, and body mass index (BMI) as covariates. The threshold chosen for the Bonferroni correction was< 7.8 × 10^−5^ (0.05/641). The effect size was used to identify the direction of the changes in the metabolite concentrations with respect to disease status. A metabolite has a positive effect size if its concentration was greater in T2D patients than in controls. Metabolites that were significantly associated with T2D in the QCBio cohort (CHD patients) but not in the QBB cohort were investigated to evaluate the interplay between T2D and CHD, and to explore the underlying biological mechanisms involved. The most significant metabolites were tested with several cardiometabolic traits: Glucose, HbA1C, and lipid traits (LDL, HDL, Triglycerides, and Total Cholesterol). This analysis was performed only in the QBB cohort because the tested traits were not available in the QCBio cohort. Alongside Bonferroni correction, we applied the Benjamini–Hochberg False Discovery Rate (FDR) procedure at a threshold of 0.05. This method calculates an adjusted value *q_i_*for each metabolite, representing the expected proportion of false positives among discoveries up to the *i^th^* ranked test. Unlike the conservative Bonferroni threshold (0.05/641 ∼ 7.8 × 10^−5^), which controls family-wise error, FDR is more flexible and allows greater power to detect true associations while still limiting false positives (48).

### 2.4 Machine learning-based predictive modeling

**Figure 2:**
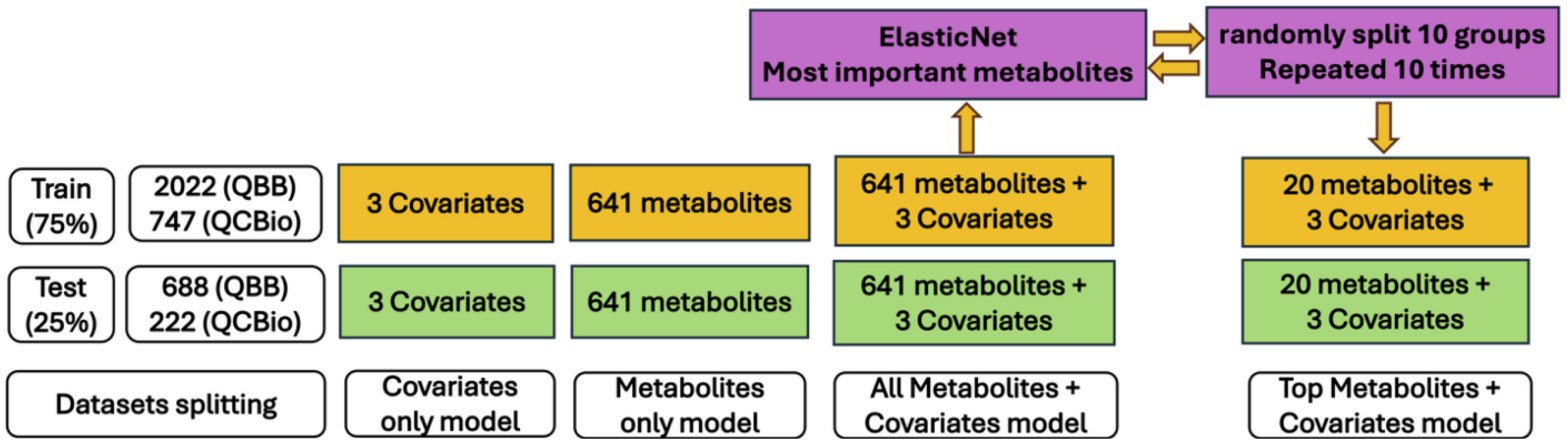
General overview of dataset splitting and different settings of models’ prediction.

Machine learning (ML) models were used to predict the occurrence of T2D. Random Forest (RF), support vector machine (SVM), extreme gradient boosting (XGBoost), and linear discriminant analysis (LDA) methods were used. Details on the selection criteria for the ML models and the rationale behind the chosen settings can be found in the **Supplementary Material**. For both cohorts, the prediction was based on four settings (**Figure 2**):

- Covariates only: The model included only age, sex, and BMI.
- Metabolites only: The model included only the 641 metabolites.
- Metabolites + covariates: The model included both covariates and metabolites.
- Top metabolites + covariates: The model included the 20 most significant metabolites plus the covariates.

For all ML experiments, the samples were divided randomly into a training set (75%) and a testing set (25%). Ten-fold cross-validation was used in all ML models where all individuals in the training set were randomly splitted into ten equal-sized groups. Each group was treated separately as a test set, and the analysis was repeated ten times. The final predictive power was evaluated based on the average accuracy and the area under the curve (AUC) for all ten predictive models. For the potential implementation of these ML models in healthcare, selecting fewer metabolites in an ML model with good predictive power is more practical and cost efficient. Hence, a feature selection technique was applied to select the most important metabolites while preserving the performance of the ML model. ElasticNet (49), which is a combination of two regularization techniques, L2 regularization (used in ridge regression) and L1 regularization (used in LASSO), has been shown to outperform LASSO when the data are highly correlated. We followed the Zou and Hastie (49) procedure for feature selection using 10-fold cross-validation. The most important metabolites were used in the ML model, and the performance was assessed (top metabolites + covariates). In this process, we avoid overfitting by running ElasticNet method on the training dataset for all 641 metabolites and extracted the most important metabolites related to T2D. Then, the performance of this model was evaluated using these important metabolites in the testing dataset. Furthermore, to avoid bias due to imbalanced classes (e.g., the number of T2D patients was much smaller than that of non-T2D individuals), especially in the QBB cohort, we performed additional analyses by reducing the sample size of non-T2D individuals (100 random selections) and assessed the performance of the ML models using the average accuracy across 100 runs. In addition, we compared models trained with Synthetic Minority Over-sampling Technique (SMOTE) (50) to those trained without SMOTE (i.e., using the 100 random downsampling runs) on the QBB cohort. In general, SMOTE is a technique used to address severe class imbalance by generating synthetic minority samples. This allowed us to evaluate whether synthetic oversampling improved predictive performance or introduced potential overfitting due to the large class imbalance. The details of how we tackled the imbalanced dataset can be found in the **Supplementary Material**.

### 2.5 Metabolite correlation and pathway enrichment analysis

Pearson’s correlation (R) was utilized to measure the strength of correlation between the most significant metabolites. Pathway enrichment analysis was performed using MetaboAnalyst 6.0 (51) (https://www.metaboanalyst.ca/) to determine the biological pathways that are highly enriched in a metabolite list than would be anticipated in random setting. For compound matching purposes, we utilized the MetaboAnalyst 6.0 software to match the Human Metabolome Database IDs with the provided compounds. Three analyses were performed, selecting the significant metabolites after Bonferroni correction in 1) the QCBio dataset, 2) the QBB dataset, and 3) the metabolites that were associated with T2D in both cohorts. Metabolite pathways with False Discovery Rate (FDR) and P< 0.05 were selected.

### 2.6 Metabolite risk score analysis

We calculated the metabolite risk scores (MRSs) using the top 20 most important metabolites derived from ElasticNet algorithm. The ElasticNet approach will use the 641 metabolites and rank them based on their importance to predict T2D. MRS is calculated as an aggregated average of metabolite values and their pre-defined effect sizes. The effect sizes are estimated using ElasticNet in the training dataset, and evaluation of MRS performance is assessed in the testing dataset. AUC, OR per 1 SD increase, and OR for top decile/quintile vs the remaining deciles/quintiles are used as performance metrics. In addition, we developed two MRS scores. One in QCBio cohort and tested in the QBB cohort and the other one developed in QBB and evaluated in QCBio.

### 2.7 Code availability

The analysis was performed in R and Python, and the code is available upon request.

## 3 Results

### 3.1 Univariate analysis

In the QCBio cohort, 42 out of 641 metabolites were significantly different between T2D patients and non-T2D patients at P < $10^{-8}$ (**Table 2**). These metabolites included 20 lipids, 10 amino acids, 8 carbohydrates, 2 peptides, and 2 xenobiotics. Twenty-seven metabolites (64.3%) were increased in T2D, and 15 metabolites (35.7%) were decreased. Thirty-six metabolites were replicated in the QBB cohort using the Bonferroni threshold (same effect size direction and P < 7.8 × $10^{-5}$) (**Table 2**). When applying the FDR threshold of 0.05, 40 metabolites were replicated, leaving only glucuronate and N6-carboxymethyllysine as nonsignificant. This demonstrates that while the strict Bonferroni criterion identifies a robust subset of associations, the FDR procedure, by accounting for correlation structure among metabolites, recovers additional biologically consistent signals. Among the replicated metabolites in QBB, 1,5-anhydroglucitol (1,5-AG) (P = 1.33 × $10^{-68}$ [-5.20, -4.16] in QBB), glucose (P = 7.14 × $10^{-57}$ [4.09, 5.23] in QBB), mannose (P = 2.61 × $10^{-54}$ [2.68, 3.45] in QBB), methylsuccinoylcarnitine (P = 1.2 × $10^{-45}$ [1.22, 1.61] in QBB), and fructosyllysine (P = 1.38 × $10^{-35}$ [1.80, 2.46] in QBB) were detected (**Table 2**). The 6 non-replicated metabolites were N6-carboxymethyllysine (P = 0.38 [-0.09, 0.33]), glucuronate (P = 0.16 and β = 0.17 [-0.06, 0.41]), 2-aminoheptanoate (P = 0.015 [0.06, 0.64]), phenylacetylglutamine (P = 7.2 × $10^{-3}$ [0.04, 0.33]), prolylglycine (P = 1.72 × $10^{-3}$ [0.11, 0.42]), and ribulonate/xylulonate/lyxonate* (P = 4.14 × $10^{-4}$ [0.19, 0.69]) (**Tables 2**, **3**).

**Table 2:** The significant metabolite in the QCBio cohort with P< 10^−8^ and their corresponding results in the QBB cohort.

Biochemical		QCBio				QBB				QBB-Lipids	
		*β*	P	FDR *q_i_*	95% CI	*β*	P	FDR *q_i_*	95% CI	*β*	P
Amino Acids	methylsuccinoylcarnitine (↑)	0.74	1.09E-18	3.12E-04	[0.58 – 0.90]	1.44	1.20E-45	4.68E-04	[1.22 – 1.61]	1.41	3.53E-43
	fructosyllysine (↑)	1.28	1.29E-16	4.68E-04	[0.98 – 1.59]	2.12	1.38E-35	6.24E-04	[1.80 –.2.46]	2.04	7.03E-33
	1-carboxyethylphenylalanine (↑)	0.48	2.40E-14	6.24E-04	[0.36 – 0.60]	0.76	1.71E-19	1.56E-03	[0.59 – 0.91]	0.72	2.84E-17
	N,N,N-trimethyl-5-aminovalerate (↑)	0.96	1.09E-13.	7.02E-04	[0.71 – 1.22]	1.24	4.26E-15	3.20E-03	[0.89 – 1.50]	1.22	1.48E-14
	2-hydroxybutyrate/2-hydroxyisobutyrate(↑)	0.71	5.51E-13.	8.58E-04	[0.53 – 0.91]	0.98	1.20E-26	7.80E-04	[0.79 – 1.15]	0.95	5.40E-25
	3-methyl-2-oxovalerate (↑)	1.17	5.20E-11	1.33E-03	[0.79 – 1.47]	1.85	3.87E-19	1.64E-03	[1.27 – 1.99]	1.78	2.69E-17
	1-carboxyethylvaline (↑)	0.45	1.54E-10	1.48E-03	[0.32 – 0.59]	0.79	2.59E-18	1.79E-03	[0.62 – 0.96]	0.76	8.57E-17
	6 bromotryptophan (↓)	-1.43	5.39E-10	1.87E-03	[-1.90 – -0.99]	-2.73	4.76E-22	1.09E-03	[-3.25 – -2.15]	-2.78	2.49E-22
	3-methyl-2-oxobutyrate (↑)	1.36	3.84E-09	2.34E-03	[0.91 – 1.80]	2.52	1.27E-20	1.33E-03	[1.89 – 2.89]	2.46	3.26E-19
	4-methyl-2-oxopentanoate (↑)	0.95	2.15E-08	2.81E-03	[0.61 – 1.25]	1.51	2.50E-15	2.89E-03	[1.02 – 1.70]	1.47	3.71E-14
Carbohydrates	1,5-anhydroglucitol (1,5-AG) (↓)	-2.15	9.82E-33	7.80E-05	[-2.51 – -1.80]	-4.64	1.33E-68	7.80E-05	[-5.20 – -4.16]	-4.68	4.40E-67
	glucose (↑)	1.7	3.26E-29	1.56E-04	[1.41 – 2.00]	4.68	7.14E-57	1.56E-04	[4.09 – 5.23]	4.64	2.99E-55
	mannose (↑)	1.76	1.01E-27	2.34E-04	[1.45 – 2.09]	3.05	2.61E-54	3.12E-04	[2.68 – 3.45]	2.97	7.85E-52
	erythronate* (↑)	1.07	2.24E-11	1.09E-03	[0.77 – 1.40]	1.7	7.68E-14	3.82E-03	[1.28 – 2.17]	1.68	1.63E-13
	fructose (↑)	0.81	3.38E-11	1.17E-03	[0.57 – 1.05]	3.15	2.25E-55	2.34E-04	[2.77 – 3.56]	3.13	9.74E-54
	ribulonate/xylulonate/lyxonate* (↑)	0.72	6.62E-10	1.95E-03	[0.49 – 0.94]	0.46	4.14E-04	1.61E-02	[0.19 – 0.69]	0.46	4.41E-04
	glucuronate (↑)	0.62	2.57E-09	2.18E-03	[0.42 – 0.83]	0.17	1.63E-01	3.17E-02	[-0.06 – 0.41]	0.18	1.39E-01
	N6-carboxymethyllysine (↑)	0.37	2.24E-08	2.89E-03	[0.25 – 0.51]	0.1	3.80E-01	3.87E-02	[-0.09 – 0.33]	0.07	4.77E-01
Lipids	sphingomyelin (d18:2/24:2)* (↓)	-1.74	1.12E-13	7.80E-04	[-2.04 – -1.18]	-2.59	1.88E-21	1.17E-03	[-2.93 – -1.92]	-2.48	9.00E-20
	sphingomyelin (d18:1/20:1, d18:2/20:0)* (↓)	-2.11	1.56E-11	9.36E-04	[-2.56 – -1.40]	-2.57	3.17E-15	3.12E-03	[-3.10 – -1.88]	-2.42	8.91E-14
	3-hydroxyoctanoate (↑)	0.51	2.23E-11	1.01E-03	[0.37 – 0.67]	0.5	1.43E-09	6.86E-03	[0.34 – 0.66]	0.48	6.38E-09
	1-(1-enyl-palmitoyl)-GPC (P-16:0)* (↓)	-1.26	3.57E-11	1.25E-03	[-1.62 – -0.89]	-1.94	6.30E-15	3.28E-03	[-2.45 – -1.48]	-1.79	8.23E-13
	sphingomyelin (d18:2/24:1, d18:1/24:2)* (↓)	-1.73	8.17E-11	1.40E-03	[-2.17 – -1.17]	-2.9	1.29E-19	1.48E-03	[-3.43 – -2.22]	-2.78	2.97E-18
	sphingomyelin (d18:2/14:0, d18:1/14:1)* (↓)	-1.35	3.73E-10	1.64E-03	[-1.60 – -0.82]	-1.84	7.89E-15	3.35E-03	[-2.04 – -1.21]	-1.79	2.88E-14
	lactosyl-N-palmitoyl-sphingosine (d18:1/16:0) (↓)	-1.58	3.74E-10	1.72E-03	[-2.08 – -1.10]	-1.7	5.78E-10	6.55E-03	[-2.27 – -1.19]	-1.67	1.43E-09
	sphingomyelin (d18:1/22:2, d18:2/22:1, d16:1/24:2)* (↓)	-1.54	5.35E-10	1.79E-03	[-1.90 – -0.98]	-2.29	1.68E-16	2.34E-03	[-2.65 – -1.64]	-2.2	1.93E-15
	deoxycholic acid 12-sulfate* (↑)	0.27	1.49E-09	2.03E-03	[0.18 – 0.36]	0.29	5.25E-16	2.57E-03	[0.22 – 0.36]	0.28	9.63E-15
	3-hydroxydecanoate (↑)	0.39	2.53E-09	2.11E-03	[0.26 – 0.52]	0.66	2.90E-12	4.84E-03	[0.47 – 0.83]	0.64	1.44E-11
	1-(1-enyl-palmitoyl)-2-palmitoleoyl-GPC (P-16:0/16:1)* (↓)	-0.99	3.02E-09	2.26E-03	[-1.32 – -0.67]	-1.47	8.21E-16	2.65E-03	[-1.84 – -1.13]	-1.43	1.54E-14
	1-stearoyl-2-arachidonoyl-GPE (18:0/20:4) (↑)	0.66	4.93E-09	2.42E-03	[0.43 – 0.87]	1.02	8.36E-17	2.26E-03	[0.72 – 1.19]	0.92	1.18E-12
	sphingomyelin (d18:2/23:1)* (↓)	-1.22	5.44E-09	2.50E-03	[-1.56 – -0.78]	-1.69	3.17E-13	4.13E-03	[-2.03 – -1.18]	-1.65	6.99E-13
	1-palmitoyl-2-arachidonoyl-GPE (16:0/20:4)* (↑)	0.52	5.49E-09	2.57E-03	[0.33 – 0.68]	0.96	1.31E-23	9.36E-04	[0.72 – 1.09]	0.89	1.10E-18
	sphingomyelin (d18:2/18:1)* (↓)	-1.42	5.94E-09	2.65E-03	[-1.80 – -0.89]	-1.82	1.23E-12	4.52E-03	[-2.25 – -1.29]	-1.75	6.22E-12
	2-aminoheptanoate (↑)	0.73	1.30E-08	2.73E-03	[0.48 – 0.98]	0.35	1.53E-02	2.29E-02	[0.06 – 0.64]	0.36	1.48E-02
	sphingomyelin (d18:1/20:2, d18:2/20:1, d16:1/22:2)* (↓)	-0.91	2.72E-08	2.96E-03	[-1.17 – -0.56]	-1.38	1.47E-15	2.81E-03	[-1.62 – -0.98]	-1.36	7.68E-15
	1-(1-enyl-palmitoyl)-2-linoleoyl-GPC (P-16:0/18:2)* (↓)	-1.23	3.95E-08	3.04E-03	[-1.67 – -0.80]	-1.38	1.44E-09	6.94E-03	[-1.85 – -0.95]	-1.24	5.77E-08
	1-palmitoyl-2-docosahexaenoyl-GPE (16:0/22:6)* (↑)	0.39	5.95E-08	3.20E-03	[0.24 – 0.52]	0.51	8.32E-12	5.07E-03	[0.34 – 0.62]	0.45	6.13E-09
	1-(1-enyl-palmitoyl)-2-oleoyl-GPC (P-16:0/18:1)* (↓)	-1.15	5.99E-08	3.28E-03	[-1.57 – -0.75]	-1.69	1.19E-13	4.06E-03	[-2.16 – -1.27]	-1.56	1.73E-11
Peptides	phenylacetylglutamine (↑)	0.36	2.74E-10	1.56E-03	[0.26 – 0.48]	0.2	7.20E-03	2.10E-02	[0.04 – 0.33]	0.21	4.80E-03
	prolylglycine (↑)	0.39	4.52E-08	3.12E-03	[0.26 – 0.54]	0.25	1.72E-03	1.84E-02	[0.11 – 0.42]	0.23	4.05E-03
Xenobiotics	mannonate* (↑)	0.99	3.06E-17	3.90E-04	[0.77 – 1.23]	2.34	2.11E-47	3.90E-04	[2.04 – 2.67]	2.29	6.88E-45
	gluconate (↑)	0.92	2.02E-15	5.46E-04	[0.69 – 1.14]	1.9	2.07E-41	5.46E-04	[1.58 – 2.13]	1.88	4.03E-40

The *β* and *P* values come from the regression models (effect sizes and P-values); QBB-Lipids is the analysis that includes LDL, HDL, Total cholesterol, and Triglycerides as covariates. Underlined metabolites are not associated with Bonferroni significance threshold. * is part of the metabolite name.

**Table 3:** The list of metabolites that were exclusively significant with T2D in only one cohort. .

Biochemical	QCBio			QBB	HbA1C		Glucose			LDL		HDL	Triglyceride		Total cholesterol	
	*β*	P	*β*	P	*β*	P	*β*	P	*β*	P	*β*	P	*β*	P	*β*	P
Significant in QBB but not in QCBio																
gamma-glutamylglutamine	0.10	5.12E-01	-2.61	1.79E-20	-0.43	4.07E-02	-0.22	4.03E-19	-0.16	4.19E-02	0.04	1.35E-01	-0.48	1.87E-18	-0.33	1.01E-07
gamma-glutamylthreonine	-0.16	4.02E-01	-2.66	1.47E-17	-0.29	1.73E-01	-0.13	2.30E-07	-0.16	4.97E-03	-0.04	1.20E-01	-0.15	8.97E-03	-0.26	5.70E-05
glutamine	0.30	2.68E-01	-4.87	1.43E-18	-1.15	7.03E-03	-0.32	4.30E-10	-0.06	6.02E-01	-0.11	2.68E-02	-0.52	2.57E-06	-0.39	1.79E-03
pseudouridine	0.26	1.37E-01	-3.27	3.81E-18	-0.25	3.82E-01	-0.23	5.60E-11	-0.02	8.07E-01	-0.08	2.71E-02	0.29	1.34E-04	0.029	7.55E-01
3-(3-amino-3-carboxypropyl)uridine*	0.17	1.22E-01	-1.96	2.18E-17	-0.30	1.04E-01	-0.12	2.30E-07	0.03	5.78E-01	-0.14	2.70E-11	0.28	5.63E-09	0.005	9.23E-01
N,N,N-trimethyl-alanylproline betaine (TMAP)	0.26	6.88E-02	-2.49	3.09E-15	0.29	1.12E-01	-0.024	2.80E-01	0.24	2.47E-06	-0.29	9.92E-42	0.28	3.43E-09	0.07	1.92E-01
3-methoxytyrosine	-0.37	3.17E-02	-3.38	4.19E-25	-0.49	1.93E-02	-0.17	6.60.E-12	-0.12	4.32E-02	0.04	1.31E-01	-0.33	1.56E-09	-0.21	7.55E-04
gamma-glutamylcitrulline*	-0.28	1.53E-02	-1.98	4.18E-22	-0.34	2.24E-02	-0.11	4.20E-10	-0.008	8.46E-01	0.10	6.98E-09	-0.25	1.77E-10	-0.015	7.30E-01
choline phosphate	0.61	1.41E-02	2.99	6.55E-17	1.76	1.67E-08	0.43	1.40E-30	0.45	1.87E-07	0.05	1.82E-01	0.61	6.88E-14	0.78	7.76E-17
pyruvate	0.43	1.43E-03	2.87	4.92E-28	1.32	2.26E-08	0.61	1.40E-107	0.11	1.05E-01	-0.25	1.19E-19	1.04	8.72E-66	0.30	2.31E-05
methyl glucopyranoside (alpha + beta)	-0.12	1.04E-03	-0.74	2.62E-15	-0.02	6.97E-01	-0.022	2.50E-09	-0.009	2.33E-01	0.003	3.99E-01	0.001	8.56E-01	-0.008	3.49E-01
1-palmitoyl-GPE (16:0)	0.48	8.12E-05	1.01	1.00E-15	0.51	1.92E-05	0.16	5.20E-28	0.19	9.53E-09	0.004	7.90E-01	0.79	6.22E-156	0.53	6.10E-51
Significant in QCBio but not in QBB																
N6-carboxymethyllysine	0.37	2.24E-08	0.10	3.80E-01	0.32	7.17E-04	0.023	4.60E-02	0.04	9.22E-02	-0.02	3.56E-02	0.06	2.53E-02	0.04	1.47E-01
glucuronate	0.62	2.57E-09	0.17	1.63E-01	0.33	4.10E-03	0.16	1.80E-32	-0.02	5.89E-01	-0.03	3.88E-02	0.22	2.75E-13	0.05	1.33E-01
2-aminoheptanoate	0.73	1.30E-08	0.35	1.53E-02	0.10	4.46E-01	0.087	8.80E-08	-0.22	3.79E-09	-0.05	1.29E-03	0.005	8.93E-01	-0.26	1.31E-10
phenylacetylglutamine	0.36	2.74E-10	0.20	7.20E-03	0.25	1.06E-03	0.041	1.30E-05	-0.04	7.53E-02	0.03	1.95E-03	-0.02	3.02E-01	-0.02	5.00E-01
prolylglycine	0.39	4.52E-08	0.25	1.72E-03	0.09	2.24E-01	0.061	1.60E-10	-0.06	5.25E-03	-0.07	6.37E-15	0.25	1.22E-34	-0.03	2.67E-01
ribulonate/xylulonate/lyxonate*	0.72	6.62E-10	0.46	4.14E-04	0.19	1.06E-01	0.18	2.20E-34	-0.12	5.99E-04	0.001	9.31E-01	0.09	3.25E-03	-0.08	3.90E-02

The columns of HbA1C, Glucose, LDL, HDL, Triglycerides, and Total Cholesterol show the results of association between the listed metabolites and these cardiometabolic traits in the QBB cohort only. Underlined metabolites are not associated with Bonferroni significance threshold.

Additionally, to assess the robustness of the observed metabolites with respect to lipid profiles, we performed a univariate analysis in the QBB cohort and adjusted for LDL, HDL, Triglycerides, and Total cholesterol. The results remained largely unchanged (**Table 2**). The significance of the top 42 metabolites in the QBB cohort ranged between 1.33 × $10^{-68}$ and 6.3 × $10^{-15}$ (**Supplementary Table 1**). The 5 most significant metabolites were 1,5-anhydroglucitol (1,5-AG), glucose, fructose, mannose, andmannonate* (**Supplementary Table 1**). These 5 metabolites were all significant in the QCBio cohort, all having P < 3.38 × $10^{-11}$ and the same effect size direction (**Supplementary Table 1**). Twelve out of 42 metabolites did not pass the Bonferronithreshold (P < 7.8 × $10^{-5}$), and 6 were not nominally significant in the QCBio data (P < 0.05): N,N,N-trimethyl-alanylproline betaine (TMAP), 3-(3-amino-3-carboxypropyl)uridine*, pseudouridine, glutamine, gamma-glutamylthreonine, and gamma-glutamylglutamine (**Table 3**). All 6 metabolites showed opposite effect sizes in QBB vs QCBio (negative in the QBB cohort and positive in the QCBio cohort; **Table 3**), except for gamma-glutamylthreonine (negative effect size in both datasets).

### 3.2 Association between top metabolites and cardiometabolic traits

We tested the association between the most significant metabolites obtained in the QCBio cohort with several cardiometabolic traits: Glucose, HbA1C, LDL, HDL, Triglycerides, and total cholesterol (**Table 3** and Online **Supplementary Table 1**). Glucose was associated with 40 of the 42 metabolites after Bonferroni correction (Online **Supplementary Table 1**). The highest evidence of association was expectedly observed for carbohydrates (e.g., fructose and mannose both with P< 10^−300^, Online **Supplementary Table 1**). HbA1C was associated with 20 of the 42 metabolites, with all amino acid metabolites being significant except 6-bromotryptophan, which was nominally significant (P = 6.28 × 10^−4^; Online **Supplementary Table 1**). Only 4 out of 20 lipid metabolites were associated with HbA1C using Bonferroni significance. On the other hand, for LDL and total cholesterol, 23 and 22 metabolites were significant, respectively (Online **Supplementary Table 1**). All carbohydrates did not show significant associations for both traits (Online **Supplementary Table 1**). Only two lipid metabolites were not significantly associated with LDL and total cholesterol (deoxycholic acid 12-sulfate* and 3-hydroxydecanoate; Online **Supplementary Table 1**). LDL and total cholesterol were not associated with the 4 peptide and xenobiotics metabolites (Online **Supplementary Table 1**). Triglyceride levels were associated with 33 of the 42 metabolites across the 4 classes of metabolites (Online **Supplementary Table 1**). Interestingly for N6-carboxymethyllysine, which was associated with T2D only in the cohort with CHD patients, no significant association was observed with any of the considered cardiometabolic traits (Online **Supplementary Table 1**).

### 3.3 Metabolite correlation and pathway enrichment analysis

**Figure 3:**
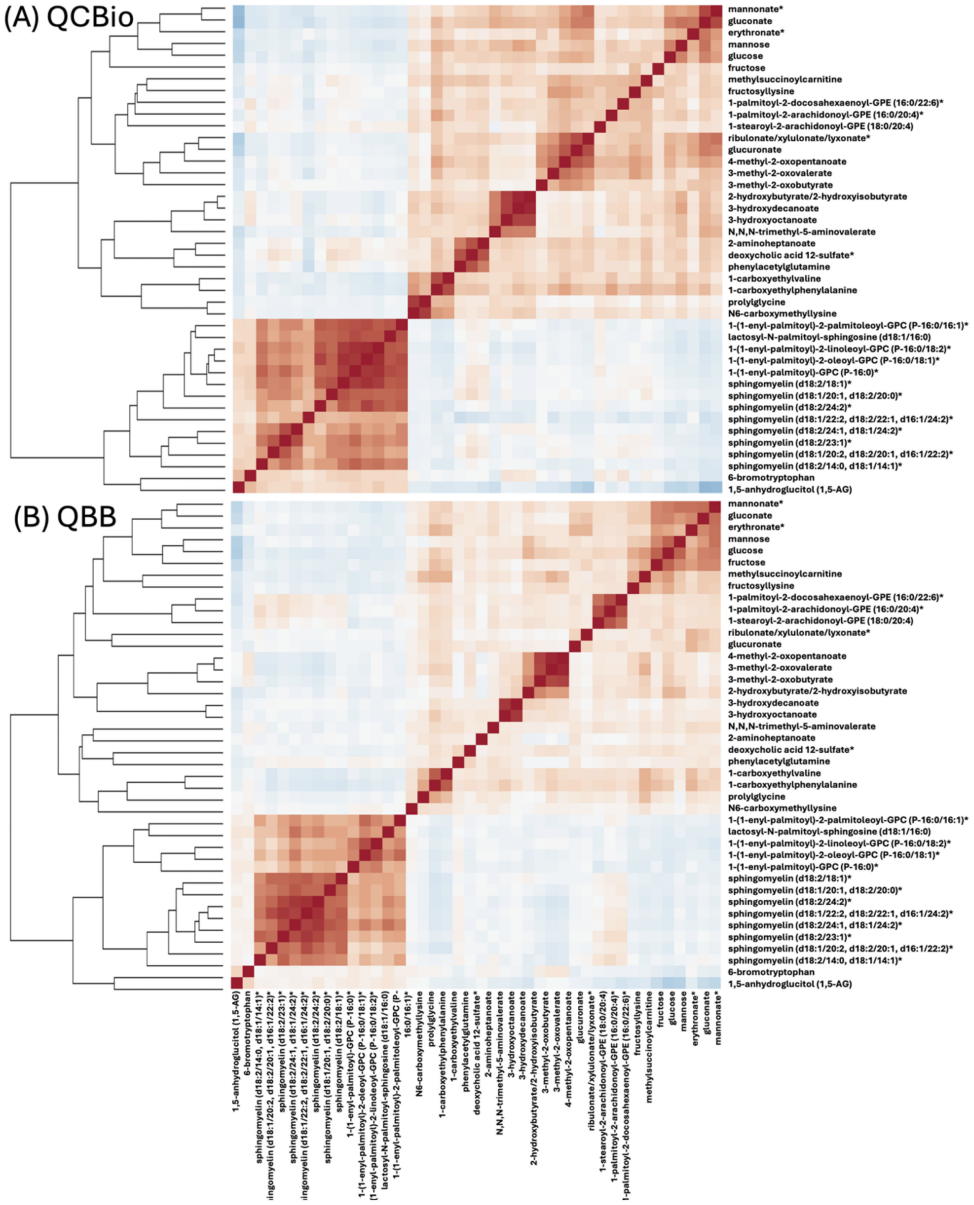
A heat map representation of Pearson’s correlation matrix of top-42 metabolites in **(A)** QCBio and **(B)** QBB cohorts. Correlations among metabolites were obtained by deriving a Pearson’s correlation coefficient between each pair of metabolites. The color scheme corresponds to correlation direction (red: positive and blue: negative).

Pearson’s correlation was calculated between the top 42 metabolites separately in QCBio and QBB cohorts (**Figure 3**). In both datasets, two main groups of metabolites with positive correlations were observed. These groups contained the same set of metabolites in both datasets (**Figure 3**). The largest group of correlated metabolites contained the 8 sphingomyelin metabolites (**Figure 3**). Mannose, glucose, and fructose were negatively correlated with 1,5-anhydroglucitol (1,5-AG) (**Figure 3**). This is concordant with the opposite direction effect sizes with T2D (e.g., increase of glucose was associated with an increased risk of T2D whereas increase of 1,5-anhydroglucitol (1,5-AG) was associated with a decreased risk of T2D) (Online **Supplementary Table 1**). Mannose, glucose, fructose, methylsuccinoylcarnitine, gluconate, mannonate*, and eryhtronate* expectedly formed a group of positively correlated metabolites.

Five pathways were obtained in the QCBio dataset **Table 4**). The most significant pathway was Valine, leucine and isoleucine biosynthesis (FDR P = 1.69 × 10^−4^). Galactose metabolism, Valine, leucine and isoleucine degradation, Fructose and mannose metabolism, and Pantothenate and CoA biosynthesis were also significant with FDR P< 0.05. The results from the QCBio and QBB common metabolites yielded 3 significant pathways, which were also present in the QCBio analysis (Valine, leucine and isoleucine degradation and biosynthesis, and galactose metabolism; **Table 4**). In the QBB dataset, only Valine, leucine and isoleucine biosynthesis was common with previous analysis (**Table 4**). Arginine biosynthesis, Alanine, aspartate and glutamate metabolism, Glyoxylate and dicarboxylate metabolism, and Glycerophospholipid metabolism were the remaining significant pathways (FDR P< 0.05; **Table 4**).

**Table 4:** Pathway enrichment analysis using MetaboAnalyst 6.0.

Metabolite Set	Total	Hits	FDR P
QCBio			
Valine, leucine and isoleucine biosynthesis	8	4	1.69E-04
Galactose metabolism	27	4	0.0178
Valine, leucine and isoleucine degradation	39	4	0.0395
Fructose and mannose metabolism	20	3	0.0395
Pantothenate and CoA biosynthesis	20	3	0.0395
QBB			
Valine, leucine and isoleucine biosynthesis	8	5	3.91E-05
Arginine biosynthesis	14	6	3.91E-05
Alanine, aspartate and glutamate metabolism	28	6	0.00242
Glyoxylate and dicarboxylate metabolism	31	5	0.0296
Glycerophospholipid metabolism	36	5	0.0472
QCBio + QBB			
Valine, leucine and isoleucine biosynthesis	8	4	2.36E-05
Valine, leucine and isoleucine degradation	39	4	0.0118
Galactose metabolism	27	3	0.0411

Total: the number of metabolites in the set; Hits: the number of metabolites intersecting with our top metabolites and the metabolite set; FDR P: the FDR corrected P value; QCBio + QBB signifies the analysis that included metabolites observed in both cohorts; Colors shows the common results between analyses.

### 3.4 Sensitivity analysis and robustness

Although age was added as a covariate to mitigate the age difference between T2D patients and controls in the QBB cohort, we ran further analysis by selecting an age matched non-T2D set from the QBB cohort. Forty-one of the top 42 metabolites identified in the full data analysis remained significant (**Supplementary Table 1**). Additionally, to further address the large sample size difference between the T2D and non-T2D groups in the QBB cohort, we randomly selected 272 non-T2D individuals from the QBB cohort and ran the analysis (with covariates) 100 times. We found that 41 metabolites were always significantly different according to the Bonferroni threshold across the 100 runs (**Supplementary Table 1**).

Furthermore, we performed another analysis on the QBB cohort that considered AGEs. Since AGEs play an important role in the pathogenesis of chronic complications of diabetes and are closely related to blood lipids, we performed univariate analysis between AGEs and LDL, HDL, and total cholesterol in the QBB cohort. Our results did not show significant differences between AGEs and lipids (**Supplementary Table 2**).

### 3.5 Machine learning-based predictive modeling

#### 3.5.1 Binary classification of T2D

**Figure 4:**
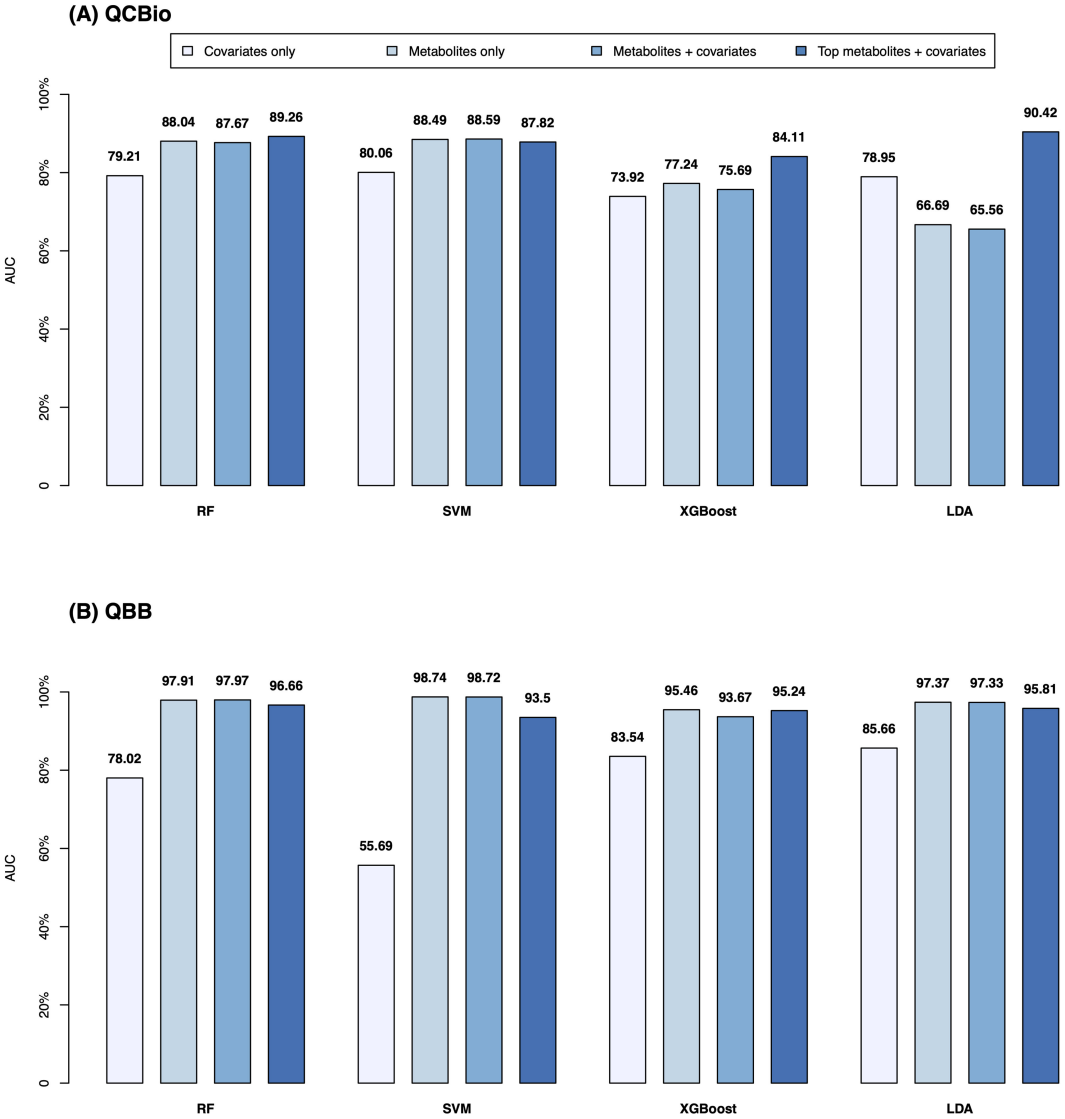
AUC of various ML models to predict T2D for 4 different settings. The ML models, RF, SVM, XGboost, and LDA are presented on the X-axis. Y-axis is the AUC. The numbers on top of each bar are the actual AUC values (in %). The bars from left to right of each model, colored as ice blue represent the Covariates only setting; light blue represent Metabolites only setting; cyan represent Metabolites and covariates setting; and dark blue represent Top 20 metabolites + covariates setting. **(A)** The AUC was computed in the QCBio cohort; and **(B)** The AUC was computed in the QBB cohort.

In the QCBio cohort, SVM showed the highest performance based on the AUC for all ML models except for the top metabolites + covariates model, where it was outperformed by LDA (AUC = 0.904 for LDA vs 0.878 for SVM; **Figure 4A**). The RF and SVM performances were similar, which made them the preferred predictive ML models. For SVM, the metabolites only, metabolites + covariates, and top metabolites + covariates models yielded similar AUCs (0.885, 0.886, and 0.878, respectively; **Figure 4A**). In the QBB cohort, similar trends were observed in both analyses (balanced and imbalanced data): adding covariates did not significantly improve the performance, choosing the top 20 metabolites showed comparable performance to the full model with a slight decrease in performance, and RF and SVM showed the highest performances (AUCs *>* 0.93; **Figure 4B**).

The performance of XGBoost and LDA was much lower than that of SVM and RF for most models, and the AUC difference with the best model exceeded 0.2 (i.e., LDA vs SVM in the QCBio cohort for the Metabolites + covariates model; **Figure 4A**). In terms of accuracy, SVM and RF for the metabolites + covariates and top metabolites + covariates models showed accuracies greater than 80% in the QCBio cohort and greater than 87% in the QBB cohort (**Supplementary Figure 2**).

In the QBB cohort, we contrasted our baseline no-SMOTE approach with models trained with SMOTE (**Supplementary Material Figure 3**). Across all four feature sets, SMOTE did not produce a systematic improvement in AUC. Without SMOTE, the leading algorithms were LDA (covariates only), SVM (metabolites only), SVM (metabolites + covariates), and RF (top metabolites + covariates). With SMOTE, the best methods shifted slightly to LDA, RF, RF, and SVM, respectively, but their AUCs were very close to those of the corresponding no-SMOTE leaders. These results indicate that our undersampling-with replication strategy provides equal or better predictive accuracy than SMOTE while limiting the risk of overfitting.

**Figure 5:**
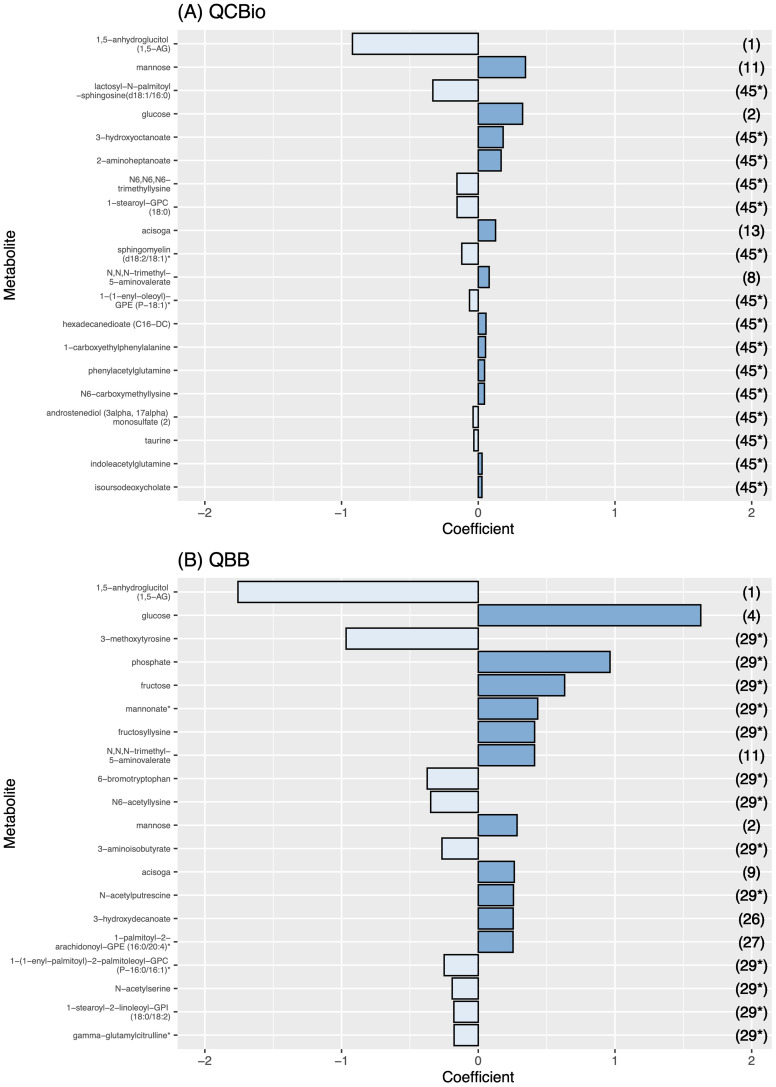
The effect size of the 20 most important metabolites for each cohort. The effect size is presented in the X-axis. Metabolite names are presented in the Y-axis. The dark blue bars represent a positive effect, while the light blue ones represent a negative effect. The numbers in the right Y-axis represent the rank of the metabolite in the other cohort. **(A)** Metabolite effect size on QCBio cohort; and **(B)** Metabolite effect size on QBB cohort.

Among the top 20 metabolites selected by the ElasticNet algorithm for both QCBio and QBB, five metabolites were common in both cohorts: 1,5-anhydroglucitol (1,5-AG), mannose, glucose, acisoga, and N,N,N-trimethyl-5-aminovalerate (**Figure 5**). Eight and nine metabolites had negative effect sizes in QCBio and QBB, respectively (**Figure 5**). The effect sizes were relatively larger in the QBB cohort (e.g., the effect size of 1,5-anhydroglucitol (1,5-AG) was -0.92 in QCBio vs -1.76 in QBB; 0.32 for glucose in QCBio and 1.63 in QBB). The use of ElasticNet to select the 20 most important metabolites did not decrease the accuracy of the ML models in predicting T2D in either cohort. We compared all ML algorithms quantitatively using the DeLong test to compare AUCs. For both cohorts, the AUCs of XGBoost were significantly different from those of other ML algorithms, whereas the AUCs of RF, SVM, and LDA were not significantly different (**Supplementary Table 3**).

### 3.6 Metabolite risk score to predict T2D

**Figure 6:**
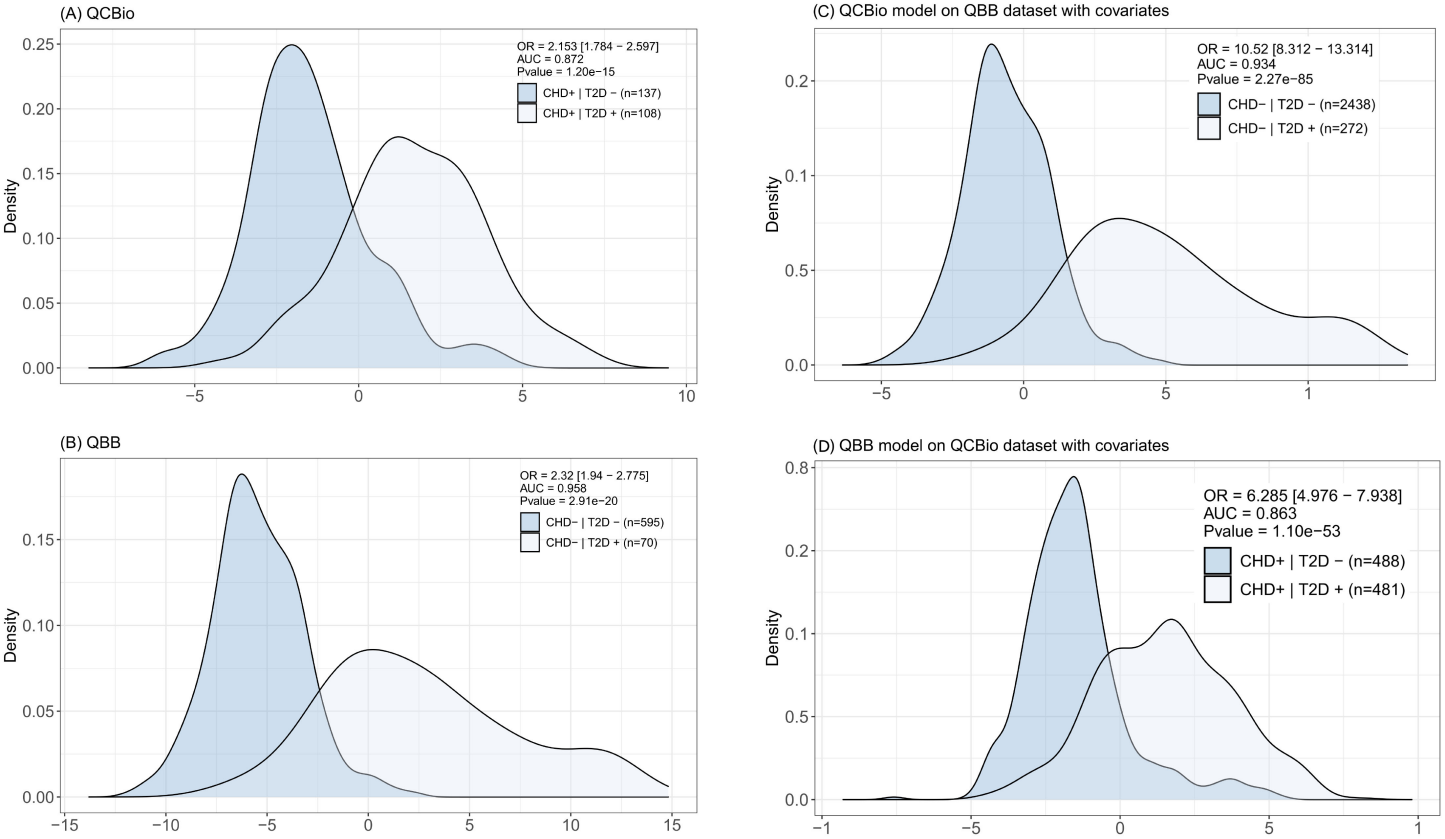
Distribution of the metabolite risk scores (MRS) for each of the reported classes. In top-right we report the OR value, AUC (0–1 scale), and the P value. For (C, D) the model is trained in one of the cohorts and tested in the other. Covariates (Gender, BMI, Age) are included in the model. **(A)** The MRS was computed in the QCBio dataset and tested in the QCBio dataset. Light blue represents the patients with both diseases (CHD+T2D+) and dark blue represents the CHD patients (CHD+T2D-); and **(B)** The MRS was computed in the QBB dataset and tested in the QBB dataset. Light blue represents the T2D patients (CHD-T2D+) and dark blue represents the healthy individuals (CHD-T2D-). **(C)** The MRS was computed in the QCBio dataset and tested in the QBB dataset. Light blue represents the T2D patients (CHD-T2D+) and dark blue represents the healthy individuals (CHD-T2D-); and **(D)** The MRS was computed in the QBB dataset and tested in the QCBio dataset. Light blue represents the patients with both diseases (CHD+T2D+) and dark blue represents the CHD patients (CHD+T2D-).

In each cohort, one model was developed in the training dataset and evaluated in the testing dataset. Both models performed well in discriminating T2D patients. The odds ratios (ORs) were 2.153 (P = 1.2 × $10^{-15}$) and 2.32 (P = 2.91 × $10^{-20}$) for QCBio and QBB, respectively (**Figures 6A, B**). The AUC was greater in QBB (0.958 for QBB vs 0.872 for QCBio; **Figures 6A, B**). We also developed an MRS in one cohort and evaluated its performance in the other cohort. The MRSdeveloped in QCBio and tested in QBB ( $MRS_{qcbio}$) performed much better than the MRS developed in QBB and tested in QCBio ( $MRS_{qbb}$) (**Figures 6C, D**). The OR for $MRS_{qcbio}$ was 10.52 (AUC = 0.934, P = 2.27 × $10^{-85}$; Figure fFig: MRS C). The OR for $MRS_{qbb}$ was 6.285 (AUC = 0.863, P = 1.1 × $10^{-53}$; **Figure 6D**). To evaluate the robustness of the $MRS_{qcbio}$ results with respect to the lower number of T2D+ |CHD- individuals (N = 272) compared to that of T2D- |CHD- individuals (N = 2,438), we selected 272 individuals from the T2D- |CHD- group and tested $MRS_{qcbio}$. The performance improved, and the OR was 19.334 [11.921 - 31.357] (**Supplementary Figure 4**). The OR of the top quintile vs remaining quintiles of $MRS_{qbb}$ tested in the QCBio data was 24.77 (P = 4.58 × $10^{-54}$) (**Supplementary Table 4**). Adjusting the model for HbA1C decreased the OR to 21.18 (**Supplementary Table 4**). Removing the 3 metabolites with the highest coefficients (i.e., 1,5-anhydroglucitol (1,5-AG), mannose, and glucose) led to ORs of 5.96 and 9.26 when accounting for HbA1C vs not accounting for HbA1C, respectively (**Supplementary Table 4**). After splitting by deciles, the ORs were expectedly greater than those after splitting by quintiles (OR = 31.87 for the decile model vs 24.77 for the quintile model) (**Supplementary Table 4**). The list of metabolites that were used for $MRS_{qcbio}$ and $MRS_{qbb}$ and their effect sizes are shown in **Table 5**. Furthermore, we ran an extra analysis to test the association between risk scores and HbA1C in non-diabetic individuals. The aim of this analysis was to determine whether these risk scores could be valuable for predicting the latent variable that is commonly used to define T2D. Hence, we used the QBB dataset after removing T2D patients (2,438 individuals). Then, we ran regression analysis between MRS and HbA1C in non-T2D patients including covariates (sex, age, and BMI). The analysis revealed a significant association (P = 3.09 × $10^{-7}$) and a positive correlation between HbA1C and risk scores (data 345 not shown).

**Table 5:** The list of top 20 metabolites in the metabolite risk score developed in QCBio and QBB.

*MRS_qcbio_*		*MRS_qbb_*	
Metabolite name	Effect size	Metabolite name	Effect size
1,5-anhydroglucitol (1,5-AG)	-0.920	1,5-anhydroglucitol (1,5-AG)	-1.758
lactosyl-N-palmitoyl-sphingosine(d18:1/16:0)	-0.332	3-methoxytyrosine	-0.967
N6,N6,N6-trimethyllysine	-0.156	6-bromotryptophan	-0.373
1-stearoyl-GPC (18:0)	-0.155	N6-acetyllysine	-0.348
sphingomyelin (d18:2/18:1)*	-0.120	3-aminoisobutyrate	-0.265
1-(1-enyl-oleoyl)-GPE (P-18:1)*	-0.064	1-(1-enyl-palmitoyl)-2-palmitoleoyl-GPC (P-16:0/16:1)*	-0.249
androstenediol (3alpha, 17alpha) monosulfate (2)	-0.038	N-acetylserine	-0.191
taurine	-0.032	1-stearoyl-2-linoleoyl-GPI (18:0/18:2)	-0.178
isoursodeoxycholate	0.027	gamma-glutamylcitrulline*	-0.177
indoleacetylglutamine	0.028	1-palmitoyl-2-arachidonoyl-GPE (16:0/20:4)*	0.254
N6-carboxymethyllysine	0.045	3-hydroxydecanoate	0.255
phenylacetylglutamine	0.046	N-acetylputrescine	0.257
1-carboxyethylphenylalanine	0.053	acisoga	0.264
hexadecanedioate (C16-DC)	0.056	mannose	0.284
N,N,N-trimethyl-5-aminovalerate	0.080	N,N,N-trimethyl-5-aminovalerate	0.412
acisoga	0.127	fructosyllysine	0.412
2-aminoheptanoate	0.167	mannonate*	0.435
3-hydroxyoctanoate	0.182	fructose	0.632
glucose	0.324	phosphate	0.964
mannose	0.345	glucose	1.628

* is part of the metabolite name.

## 4 Discussion

We studied the metabolomic signatures of T2D patients stratified by the presence or absence of CHD in a Middle Eastern cohorts from the Qatar BioBank and Qatar Cardiovascular Biorepository. Univariate analysis was performed to identify metabolites that were differentially expressed between T2D patients who had CHD and T2D patients without CHD. In addition, matching and resampling methods were used to tackle the cohorts class imbalance problem. Pathway enrichment analysis was utilized to observe the significant metabolic alterations on a pathway level related to T2D, in the presence and absence of CHD. ML modeling was used to predict T2D in the presence and absence of CHD. Metabolite risk scores were developed and showed great discriminative power for T2D, especially in the CHD cohort. This study is important for dissecting the metabolomics signatures of two metabolic diseases that are biologically interlinked. This study sheds light on metabolites that behave differently between T2D patients and non T2D patients with respect to CHD status. Delaying or preventing CHD in T2D patients can have a major clinical impact.

In our study, we compared several ML models that included all metabolites and covariates, and we also developed a model that only included the top metabolites. The aim of this model was to assess the performance of using a smaller set of metabolites as a practical and cost-effective choice in clinical practice. Our results provide strong evidence that many metabolites are altered in T2D patients with CHD. Therefore, this small panel of metabolites can be used as a diagnostic/predictive tool after further clinical validation. The discovered metabolites could be further investigated for therapeutic interventions to reduce the incidence of CHD in T2D patients.

Our study identified several previously reported metabolites that are associated with T2D. These metabolites were replicated in both the QCBio and QBB cohorts. 1,5-anhydroglucitol (1,5-AG) was the most significant metabolite in both cohorts. This metabolite has been identified as a marker for glycemic control (38, 52, 53). The metabolite was decreased in T2D patients, but the decrease in the cohort without CHD was two-fold lower than that in the cohort with CHD. Other known carbohydrates, including glucose, mannose, and fructose, were associated with T2D in both cohorts.

We investigated metabolites that were associated with T2D in QCBio (CHD cohort) but not in QBB. Since the size of the QCBio dataset is much smaller (one-third) than the QBB dataset (**Supplementary Figure 1**), the identification of such metabolites is unlikely because of the lower statistical power of QCBio. One of these metabolites is N6-carboxymethyllysine (CML), which is a type of advanced glycation end product (AGE) that is commonly used as a marker for analyzing AGEs in food (54, 55). AGEs are a group of bioactive molecules that result from nonenzymatic glycation of proteins, lipids, and nucleic acids and are associated with the progression of degenerative diseases such as diabetes and atherosclerosis (56, 57). AGEs may also contribute to vascular complications in T2D patients (58). In patients with CHD, elevated serum AGEs have been reported even without any comorbidities, such as T2D (59). Increased CML levels are known to be associated with arterial stiffness (60). In the present study, CML levels were found to be significantly associated with CHD in individuals with T2D, while mean CML levels were similar between CHD patients and non-CHD patients without T2D. Moreover, the levels of CML increased significantly in patients with both CHD and T2D (**Supplementary Figure 5**).

Phenylacetylglutamine (PAGln) was also significant in the QCBio cohort but not in the QBB cohort. Recently, PAGln was identified as a novel metabolic biomarker for ischemic stroke (61). PAGln is a gut microbiota-derived metabolite that may induce cardiovascular events by activating platelets and increasing the risk of thrombosis (62). The PAGln level appeared to be significantly increased in T2D patients in recent studies (63, 64). In our study, the levels of PAGln were increased in both the QCBio and QBB cohorts for T2D patients, but the highest levels were observed in T2D patients who had CHD (**Supplementary Figure 5**). These findings are consistent with the results of Nemet et al. (63), who indicated that elevated levels of PAGln can predict the occurrence of adverse cardiac events such as heart attack and stroke in patients with T2D.

Another metabolite that showed statistical significance in the CHD cohort but not in the QBB cohort was glutamine. Glutamine is an amino acid that plays a significant role in the biosynthesis of proteins. Glutamine deficiency is associated with many conditions, including type 2 diabetes (65, 66) and insulin resistance (67, 68), which are considered risk factors for CVD (69). In our study, glutamine levels were reduced in T2D patients compared with non-T2D patients in the QCBio cohort, in accordance with previous findings (66, 70–72). Glutamine serves as an L-arginine precursor for the production of nitric oxide and mitigates risk factors for CVD (73). A recent research proposal aimed at investigating the hypothesis that targeting glutamine-dependent pathways in monocytes/macrophages may limit the inflammatory phenotype and cardiovascular events in diabetic patients (https://anr.fr/Project-ANR-19-CE17-0030). Since gamma-glutamylthreonine and gamma-glutamylglutamine are associated with pathways involving glutamine as a substrate, their levels expectedly showed similar trends in our data.

Our study showed results consistent with those of metabolomics studies of T2D in other regions of the world. For example, our analysis showed that the 1,5-anhydroglucitol carbohydrate content was significantly decreased in T2D patients, regardless of CHD status, which is consistent with the findings of Suhre et al. (37), who studied the German population. Similar observations were observed for increased levels of glucose, mannose, 3-methyl-2-oxovalerate, and erythronate in T2D patients. An identical trend was observed in a Chinese cohort for the glucose metabolite, where its level was increased in T2D patients who also had CHD (74). Another study (75) confirmed previous observations and showed a significant increase in glucose metabolite levels in T2D patients with CHD in a Chinese population. A study (76) of a Malaysian cohort showed that N6-carboxymethyllysine metabolite levels were significantly increased in diabetic and ischemic heart disease (IHD) patients compared with those in T2D patients but not in IHD patients, which matches our findings, where these metabolite levels increased in diabetic CHD patients compared with those in T2D patients without CHD.

Furthermore, we investigated the CHD-specific metabolites, where we compared the metabolites from non-T2D patients in QBB and non-T2D CHD patients in QCBio. CHD-related metabolites were identified and were consistent with the literature including ornithine, 3-amino-2-piperidone, Sphingosine-1-phosphate (S1P), aspartate (see Online **Table 2**). For instance, Virak et al. (77), indicated that the citrulline-to-ornithine ratio is a critical risk factor for HF and CHD. Similar findings have been observed in other studies (78, 79). As a consequence, any disturbance in the ornithine cycle causes increase of the 3-amino-2-piperidone levels, which damages cardiovascular system (80). Previous study showed that the Sphingosine-1-phosphate plays an important role in the occurrence and development of many cardiovascular diseases (81–84). Numerous studies investigated the association between aspartate metabolite and CVD diseases (85–87). They revealed that elevated aspartate level may indicate an increased CVD risk (85–87).

Pathway enrichment analysis showed that the galactose metabolism was, as expected, significantly associated with T2D in both cohorts. This has been observed in previous studies (38, 88, 89). Leucine, isoleucine, and valine, which are branched-chain amino acids (BCAAs), also showed significant association with T2D in both cohorts (**Table 4**). There has been consensus that this class of amino acids is among the strongest biomarkers of T2D as well as other pathogenesis metabolic disturbances in obesity and cardiovascular diseases (90–92). Also, Starch and sucrose metabolism was among the top significant metabolisms in both cohorts (**Table 4**), which is in accordance with many previous studies (93–95). For instance, Sun et al. (95) performed metabolic pathway analysis with the MetPA tool, and they found that starch and sucrose metabolism was one of the potential biomarkers for T2D. Furthermore, Arginine biosynthesis was found associated with T2D in the QBB cohort only. Arginine is a precursor for nitric oxide, and was shown to be reduced in patients with T2D due to a decreased conversion of arginine to nitric oxide (96). Moreover, the long-term oral L-Arginine administration showed an improvement of hepatic insulin in patients with T2D (97). This validates our findings and adds evidence to the importance of Arginine in the pathophysiology of T2D. The non-significance of Arginine biosynthesis pathway in our CHD cohort might be due to a reduced sample size, or due to the disruption of Arginine metabolite with the CHD cohort as shown on the same data previously (40).

ML models were applied to predict T2D in the presence and absence of CHD using metabolites and demographic data. These models account for nonlinear relationships between metabolites and might yield better predictions. The F_1_ scores for predicting T2D in the QBB (non-CHD) cohort were greater than those in the QCBio cohort. The scores were *>* 74% for both the RF and SVM models for all classes. The accuracy values were relatively high and reached 80%. In the present study, we accounted for class imbalance to mitigate the potential of bias in ML results by selecting subsets of each class to make them balanced. So, we (i) established an age-matched non-T2D control group from the QBB cohort, which validated 41 of the 42 metabolites found in the complete analysis and (ii) downsized the non-T2D group (n=272) 100 times, redoing the analysis with covariates each time. In all 100 resampled datasets, 41 metabolites continued to be significant following the Bonferroni correction. These outcomes show that our results are resilient to both age disparity and class disparity and are not influenced by the unequal case-control ratios across groups (**Supplementary Material**). For binary classification, which we used to predict T2D vs non-T2D within each cohort, selecting 20 metabolites instead of the 641 tested metabolites led to similar performance (similar AUC and similar accuracy). The RF and SVM models exhibited similar performances and outperformed the XGBoost and LDA models. The RF model in the QBB showed an AUC of 0.96 for the top metabolites + covariates model, which was greater than the AUC in QCBio (0.89). Since CHD is a cardiometabolic disorder, metabolism is disrupted and might overlap with T2D metabolism disruption, which makes the prediction of T2D in CHD patients slightly more difficult.

Metabolite risk scores were also developed to predict T2D using the QCBio and QBB cohorts. MRS assumes linear relationships between metabolites and can be easily integrated in clinical practice. It is calculated as a weighted sum of metabolites and prespecified coefficients. Similar MRSs were developed previously in 4 Finnish cohorts using 3 metabolites (98). In the Finnish study, the top 20% of individuals with respect to their MRS had a ten-fold increased risk of developing T2D, and the OR per 1 standard deviation (SD) increase was 1.76. Our MRS comprised 20 metabolites, which is 17 more metabolites than in the Finnish study. In our study, the OR per 1 SD increase was 10.52 in *MRS_qcbio_*, which was developed in the CHD cohort (QCBio). Individuals in the top *MRS_qcbio_*quintile had a 24.77-fold increased risk of developing T2D. Adjusting for HbA1C reduced the performance to an OR of 21.18. Removing the most important metabolites from *MRS_qcbio_*, such as glucose and mannose, and adjusting for HbA1C led to an OR of 13.19 for the top quintile. *MRS_qcbio_*performed better than *MRS_qbb_*in predicting T2D, potentially because T2D patients are better defined in the QCBio cohort, and they are older. *MRS*_−_*_seq_*can eventually be used in clinical practice with other types of risk scores for better interventions and treatments of individuals at high risk of developing T2D. However, these MRSs need validation in a new independent cohort to replicate our findings. Metabolic risk scores are dynamic and modifiable, unlike genetic risk scores. They complement existing genetic and clinical risk scores and can be an alternative in the absence of the other scores. They are particularly helpful for preclinical disease stages and hold promise for early detection. Genetic risk scores are fixed over time, and do not reflect recent changes in the biological process. Implementation of MRS, and other types of risk scores, is crucial for personalized prevention and treatment plans, but face difficulties. All scores need to be well-validated, ultimately across ancestral groups. Moreover, healthcare systems should be equipped with state-of-art techniques that generate the various omics data that is required to build all these scores.

Our study has a few limitations. First, there was an imbalance of the different disease combinations, where the number of T2D patients was relatively smaller in the QBB cohort. A cohort of well-defined T2D patients is needed to confirm our results and increase the sample size. A longitudinal design would be preferable to a retrospective design. T2D patients could be followed up over time to assess CHD incidence and link it to metabolomics data, which should be generated at multiple time points. This optimal study design raises challenges about the cost of generating multiple metabolomics datasets, also the reproducibility and accuracy of metabolomics data generation, which might vary because of data generation artifacts. There have been proposed approaches to deal with systematic differences across metabolomics datasets, which can be used to improve reproducibility of our results (99). The results from this study might also be affected by the statistical power and low sample size of some disease groups. Therefore, the validation and reproducibility of our results should be explored in future studies from the same population, ideally with larger sample size. A well-designed study with well-defined T2D and CHD cases is likely to yield more robust results, even with smaller sample sizes and therefore lower cost.

Second, although QBB and QCBio cohorts were different in terms of age, we tried to mitigate for the age difference impact on the resulting metabolites by adding age as covariate in all regression models. Ideally, both cohorts should be matched on sex and age, but because of small sample size, especially for T2D patients in QBB, this was difficult to achieve in our study. Age at onset is another important variable that could be checked with respect to obtained metabolites, but this variable was absent in our data. Furthermore, while Age, BMI and Gender, were already included as covariates, we did not consider cultural, dietary or genetic factors, which could bring new important insights to our study.

Third, all carried analysis has been done on datasets that belong to the same Qatari population. This limits our generalizability claims. Hence, we are in the process of generating new independent metabolomics data, which will be used to validate these scores, however that will take some time. In addition, our plan is to include other datasets from the Gulf region like Saudi Arabia for generalizability purposes.

## 5 Conclusion

In this study, focusing on circulating metabolites in Middle Eastern cohorts from the QCBio and QBB, we identified and replicated metabolites that are associated with T2D. Several metabolites associated with T2D were identified only by stratifying for CHD status. Pathway enrichment analysis was utilized to observe the significant metabolic pathways that were associated with T2D in the presence and absence of CHD. ML models were applied and showed good predictive power to predict T2D in the presence and absence of CHD. RF and SVM were the best models in our study. A metabolite risk score for the prediction of T2D was developed and showed great performance, especially the score developed from the QCBio cohort.

## Data Availability

The original contributions presented in the study are included in the article/**Supplementary Material**. Further inquiries can be directed to the corresponding author/s.
