# Supplementary material for: Coronary heart disease and type 2 diabetes metabolomic signatures in the Middle East
Source: Front Endocrinol (Lausanne). 2025 Nov 17;16:1531525. doi: 10.3389/fendo.2025.1531525 (PMC12665572; doi:10.3389/fendo.2025.1531525)
Supplement: Supplementary file 1 [file DataSheet1.pdf]

Coronary Heart Disease and Type 2 Diabetes Metabolomic Signatures in the Middle East

1 SUPPLEMENTARY MATERIAL

1.1 Metabolomics profiling details

Normalization and data processing: We used Metabolons's standard data processing pipeline. First, raw area counts were normalized to correct for inter-day instrument variability by registering the medians of each run day batch to equal 1. Then, each metabolite's values within a batch were normalized proportionally. Hence, the median across all samples in the batch equaled 1. After that data from all batches were combined into a unified dataset. Following the batch correction, log transformation (log10) applied to stabilize the variance and approximate normality.

QC checks for residual batch effects: We evaluated the effectiveness of normalization by performing unsupervised principal component analysis (PCA) and hierarchical clustering. These analyses showed that samples did not cluster by batch or run date, indicating that batch-related variation had been adequately removed.

Metabolite identification: Metabolon identifies metabolites using their proprietary library of purified standards and retention indices, accurate mass data, and MS/MS fragmentation spectra. Metabolite identities are confirmed by comparison to authenticated standards when available (Level 1 identification, MSI criteria). For compounds where standards are not available, identifications are based on spectral library matches (putative annotations, Level 2).

1.2 Machine learning in metabolomics

In general, metabolomic studies can be categorized into three branches: targeted analysis Shulaev (2006); Griffiths and Wang (2009), untargeted analysis (metabolite profiling) Fiehn (2002); Halket et al. (2005), and metabolic fingerprinting (exometabolomics) Allen et al. (2003); Mapelli et al. (2008); Silva and Northen (2015); Thomas et al. (2019). Since, our current study falls in the second category, i.e., untargeted analysis, where we have hundreds of metabolites with a complex interaction of data. This necessitates the utilization of advanced computational methods, especially machine learning (ML) algorithms, to analyze and extract meaningful information out of the dataset. However, determining the type of ML algorithm to be utilized depends on multiple factors, including the type of dataset, the research question that needs to be answered, the ML category used. Accordingly, our study falls under supervised machine learning category, as our problem involves inferring a function from a labeled instances in the dataset and we expect a specific output, which is a predicted label. In addition, since our fundamental analysis can be casted as a classification problem with two categories. Hence, we used logistic regression for this purpose. Moreover, we carried out meta-analysis and chose Random Forest (RF), Support Vector Machines (SVM), extreme Gradient Boosting (XGBoost), and Linear Discriminant Analysis (LDA) methods. The reason behind choosing these methods is based on the literature review we conducted on the best ML methods used for metabolomics analysis, where we find Galal et al. (2022); Mendez et al. (2019); Vu et al. (2019). For example, Mendez et al. Mendez et al. (2019) carried a comprehensive comparative study between eight ML algorithms for ten clinical metabolomic datasets and found that SVM among the best ML algorithms across all metabolomic datasets. In another study Trainor et al. (2017), it evaluated the performance of

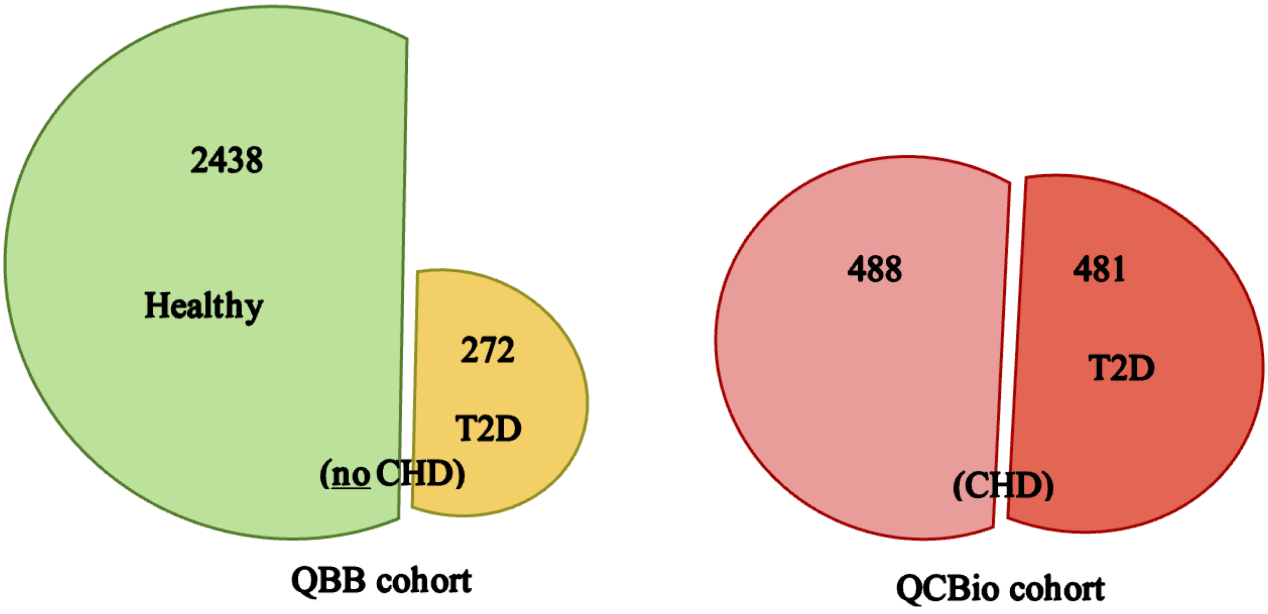

Figure S1. General description of both cohorts. In the QBB cohort (control individuals), all individuals had no CHD and approximately 10% had T2D. In the QCBio cohort (CHD patients), all individuals had CHD and approximately 50% had T2D.

seven classification algorithms and demonstrated the superiority of SVM and RF classifiers. Additionally, in the literature we found these methods are the most utilized ones Han et al. (2021). For instance, RF models have been exploited in many studies Han et al. (2021); Li et al. (2021); Oh et al. (2020). Similar observation with SVM method Liu et al. (2016, 2021), LDA model Liu et al. (2021); Wallace et al. (2020), and Logistic Regression method Liu et al. (2021); Kouznetsova et al. (2019); Jung et al. (2021). As per the metrics to assess the classification problem, the commonly used ones are accuracy Gajda and Chlebus (2022) as well as area under the curve (AUC) Gajda and Chlebus (2022); Yala et al. (2019).

1.3 Statistical Analysis Software

For R software (version 4.1.2), we used the following packages: dplyr (1.1.4), datasets (4.5.1), caret (7.0.1), randomForest (4.7-1.2), pROC (1.19.0-1), rminer (1.4.9), mlr (2.19.3), stringr (1.5.1), RColorBrewer (1.1-3), glmnet (4.1-10), doParallel (1.0.17), foreach (1.5.2), and smotefamily (1.4.0). For Python software, we utilized the following packages: scikit-learn (1.7.0), pandas (2.3.0), numpy (2.3.1), and matplotlib (3.10.3).

REFERENCES

Allen, J., Davey, H. M., Broadhurst, D., Heald, J. K., Rowland, J. J., Oliver, S. G., et al. (2003). High-throughput classification of yeast mutants for functional genomics using metabolic footprinting. *Nature biotechnology* 21, 692–696

Fiehn, O. (2002). Metabolomics—the link between genotypes and phenotypes. *Functional genomics* , 155–171

Gajda, S. and Chlebus, M. (2022). A probability-based models ranking approach: An alternative method of machine-learning model performance assessment. *Sensors* 22, 6361

Galal, A., Talal, M., and Moustafa, A. (2022). Applications of machine learning in metabolomics: Disease modeling and classification. *Frontiers in genetics* 13, 1017340

Griffiths, W. J. and Wang, Y. (2009). Mass spectrometry: from proteomics to metabolomics and lipidomics. *Chemical Society Reviews* 38, 1882–1896

Halket, J. M., Waterman, D., Przyborowska, A. M., Patel, R. K., Fraser, P. D., and Bramley, P. M. (2005). Chemical derivatization and mass spectral libraries in metabolic profiling by gc/ms and lc/ms/ms. *Journal of experimental botany* 56, 219–243

Han, S., Van Treuren, W., Fischer, C. R., Merrill, B. D., DeFelice, B. C., Sanchez, J. M., et al. (2021). A metabolomics pipeline for the mechanistic interrogation of the gut microbiome. *Nature* 595, 415–420

Jung, S., Ahn, E., Koh, S. B., Lee, S.-H., and Hwang, G.-S. (2021). Purine metabolite-based machine learning models for risk prediction, prognosis, and diagnosis of coronary artery disease. *Biomedicine & Pharmacotherapy* 139, 111621

Kouznetsova, V. L., Kim, E., Romm, E. L., Zhu, A., and Tsigelny, I. F. (2019). Recognition of early and late stages of bladder cancer using metabolites and machine learning. *Metabolomics* 15, 1–15

Li, Y., Ma, G., Shao, H., Xiao, P., Lu, J., Xu, J., et al. (2021). Electrochemical lithium storage performance of molten salt derived v 2 snc max phase. *Nano-micro letters* 13, 1–10

Liu, S., Gui, Y., Wang, M. S., Zhang, L., Xu, T., Pan, Y., et al. (2021). Serum integrative omics reveals the landscape of human diabetic kidney disease. *Molecular metabolism* 54, 101367

Liu, Y., Yieh, L., Yang, T., Drinkenburg, W., Peeters, P., Steckler, T., et al. (2016). Metabolomic biosignature differentiates melancholic depressive patients from healthy controls. *BMC genomics* 17, 1–17

Mapelli, V., Olsson, L., and Nielsen, J. (2008). Metabolic footprinting in microbiology: methods and applications in functional genomics and biotechnology. *Trends in biotechnology* 26, 490–497

Mendez, K. M., Broadhurst, D. I., and Reinke, S. N. (2019). The application of artificial neural networks in metabolomics: a historical perspective. *Metabolomics* 15, 142

Oh, T. G., Kim, S. M., Caussy, C., Fu, T., Guo, J., Bassirian, S., et al. (2020). A universal gut-microbiome-derived signature predicts cirrhosis. *Cell metabolism* 32, 878–888

Shulaev, V. (2006). Metabolomics technology and bioinformatics. *Briefings in bioinformatics* 7, 128–139

Silva, L. P. and Northen, T. R. (2015). Exometabolomics and msi: deconstructing how cells interact to transform their small molecule environment. *Current Opinion in Biotechnology* 34, 209–216

Thomas, S. C., Tamadonfar, K. O., Seymour, C. O., Lai, D., Dodsworth, J. A., Murugapiran, S. K., et al. (2019). Position-specific metabolic probing and metagenomics of microbial communities reveal conserved central carbon metabolic network activities at high temperatures. *Frontiers in microbiology* 10, 1427

Trainor, P. J., DeFilippis, A. P., and Rai, S. N. (2017). Evaluation of classifier performance for multiclass phenotype discrimination in untargeted metabolomics. *Metabolites* 7, 30

Vu, T., Siemek, P., Bhinderwala, F., Xu, Y., and Powers, R. (2019). Evaluation of multivariate classification models for analyzing nmr metabolomics data. *Journal of proteome research* 18, 3282–3294

Wallace, P. W., Conrad, C., Brückmann, S., Pang, Y., Caleiras, E., Murakami, M., et al. (2020). Metabolomics, machine learning and immunohistochemistry to predict succinate dehydrogenase mutational status in phaeochromocytomas and paragangliomas. *The Journal of pathology* 251, 378–387

Yala, A., Lehman, C., Schuster, T., Portnoi, T., and Barzilay, R. (2019). A deep learning mammography-based model for improved breast cancer risk prediction. *Radiology* 292, 60–66

		QCBio		QBB		QBB-Matched	
	Biochemical	β	P	β	P	β	P
Amino Acids	methylsuccinoylcarnitine (↑)	0.74	1.09E-18	1.44	1.20E-45	1.52	9.46E-34
	fructosyllysine (↑)	1.28	1.29E-16	2.12	1.38E-35	2.13	1.72E-28
	2-hydroxybutyrate/2-hydroxyisobutyrate (↑)	0.71	5.51E-13	0.98	1.20E-26	1.03	1.47E-20
	3-methoxytyrosine(↓)	-0.37	3.17E-02	-3.38	4.19E-25	-3.15	8.43E-21
	6-bromotryptophan (↓)	-1.43	5.39E-10	-2.73	4.76E-22	-2.52	1.98E-18
	N,N-dimethylalanine (↓)	-0.36	1.22E-05	-1.47	3.98E-21	-1.38	1.87E-18
	3-methyl-2-oxobutyrate (↑)	1.36	3.84E-09	2.52	1.27E-20	2.30	2.70E-16
	1-carboxyethylphenylalanine (↑)	0.48	2.40E-14	0.76	1.71E-19	0.85	2.03E-20
	3-methyl-2-oxovalerate (↑)	1.17	5.20E-11	1.85	3.87E-19	1.79	9.05E-16
	glutamine (↑)	0.30	2.68E-01	-4.87	1.43E-18	-4.70	2.20E-15
	1-carboxyethylvaline (↑)	0.45	1.54E-10	0.79	2.59E-18	0.86	1.48E-18
	leucine (↑)	1.46	3.30E-06	3.07	2.54E-16	2.95	4.79E-13
	4-methyl-2-oxopentanoate (↑)	0.95	2.15E-08	1.51	2.50E-15	1.43	9.18E-13
	N,N,N-trimethyl-alanylproline betaine (TMAP) (↑)	0.26	6.88E-02	-2.49	3.09E-15	-2.13	7.41E-12
	N,N,N-trimethyl-5-aminovalerate (↑)	0.96	1.09E-13	1.24	4.26E-15	1.32	9.75E-15
Carbohydrates	1,5-anhydroglucitol (1,5-AG) (↓)	-2.15	9.82E-33	-4.64	1.33E-68	-4.29	9.48E-53
	glucose (↑)	1.70	3.26E-29	4.68	7.14E-57	5.45	3.26E-44
	fructose (↑)	0.81	3.38E-11	3.15	2.25E-55	3.36	5.40E-41
	mannose (↑)	1.76	1.01E-27	3.05	2.61E-54	3.20	1.34E-40
	pyruvate (↑)	0.43	1.43E-03	2.87	4.92E-28	2.89	8.40E-23
Lipids	1-palmitoyl-2-arachidonoyl-GPE (16:0/20:4)* (↑)	0.52	5.49E-09	0.96	1.31E-23	1.10	1.50E-19
	sphingomyelin (d18:2/24:2)* (↓)	-1.74	1.12E-13	-2.59	1.88E-21	-2.51	1.45E-19
	sphingomyelin (d18:2/24:1, d18:1/24:2)* (↓)	-1.73	8.17E-11	-2.90	1.29E-19	-2.95	5.72E-19
	1-palmitoyl-2-oleoyl-GPE (16:0/18:1) (↑)	0.30	6.56E-06	0.63	2.30E-17	0.64	1.18E-13
	choline phosphate (↑)	0.61	1.41E-02	2.99	6.55E-17	3.06	1.44E-14
	1-stearoyl-2-arachidonoyl-GPE (18:0/20:4) (↑)	0.66	4.93E-09	1.02	8.36E-17	0.98	1.33E-13
	sphingomyelin (d18:1/22:2, d18:2/22:1, d16:1/24:2)* (↓)	-1.54	5.35E-10	-2.29	1.68E-16	-2.36	2.53E-16
	1-arachidonoyl-GPE (20:4n6)* (↑)	0.77	1.76E-06	1.47	2.15E-16	1.52	8.74E-15
	deoxycholic acid 12-sulfate* (↑)	0.27	1.49E-09	0.29	5.25E-16	0.31	1.07E-14
	1-(1-enyl-palmitoyl)-2-palmitoleoyl-GPC (P-16:0/16:1)* (↓)	-0.99	3.02E-09	-1.47	8.21E-16	-1.45	6.72E-14
	1-palmitoyl-GPE (16:0) (↑)	0.48	8.12E-05	1.01	1.00E-15	1.06	2.06E-13
	sphingomyelin (d18:1/20:2, d18:2/20:1, d16:1/22:2)* (↓)	-0.91	2.72E-08	-1.38	1.47E-15	-1.39	2.60E-14
	sphingomyelin (d18:1/20:1, d18:2/20:0)* (↓)	-2.11	1.56E-11	-2.57	3.17E-15	-2.72	9.39E-16
	1-(1-enyl-palmitoyl)-GPC (P-16:0)* (↓)	-1.26	3.57E-11	-1.94	6.30E-15	-1.88	1.87E-13
Nucleotide	pseudouridine (↑)	0.26	1.37E-01	-3.27	3.81E-18	-2.80	1.80E-13
	3-(3-amino-3-carboxypropyl)uridine* (↑)	0.17	1.22E-01	-1.96	2.18E-17	-1.68	8.65E-13
Peptides	gamma-glutamylcitrulline* (↓)	-0.28	1.53E-02	-1.98	4.18E-22	-1.97	8.62E-21
	gamma-glutamylglutamine (↑)	0.10	5.12E-01	-2.61	1.79E-20	-2.39	1.30E-16
	gamma-glutamylthreonine (↓)	-0.16	4.02E-01	-2.66	1.47E-17	-2.38	5.24E-14
Xenobiotics	mannonate* (↑)	0.99	3.06E-17	2.34	2.11E-47	2.53	1.98E-38
	gluconate (↑)	0.92	2.02E-15	1.90	2.07E-41	1.84	4.69E-31
	methyl glucopyranoside (alpha + beta) (↓)	-0.12	1.04E-03	-0.74	2.62E-15	-0.65	1.09E-13

Table S1. The 42 most significant metabolite in the QBB cohort and their corresponding results in the QCBio and QBB-Matched. The QBB-Matched represents the results analysis of selecting an age-matched non-T2D set from the QBB cohort. The QBB-Matched results represent the average of 100 runs of randomly selected 272 non-T2D individuals from the QBB cohort.

QBB cohort		
	β	P
Triglyceride	0.06358	0.0122
LDL	0.04030	0.133
HDL	-0.02452	0.0323
Cholesterol Total	0.04071	0.162

Table S2. Univariate analysis between AGEs and LDL, HDL, and total cholesterol in the QBB cohort.

**QCBio cohort**				
Algorithm	RF	SVM	XGBoost	LDA
RF	-	0.3416705	0.02440212	0.2708251
SVM	0.3416705	-	0.01598019	0.8327119
XGBoost	0.02440212	0.01598019	-	0.00849191
LDA	0.2708251	0.8327119	0.00849191	-
**QBB cohort**				
RF	-	0.6644548	0.00515316	0.2091238
SVM	0.6644548	-	0.00425816	0.7533963
XGBoost	0.00515316	0.00425816	-	0.00427056
LDA	0.2091238	0.7533963	0.00427056	-

Table S3. The significant difference between machine learning models using Delong test.

				Covariates = age, sex, and BMI		Covariates = age, sex, BMI, and HbA1c	
				OR	P	OR	P
Excluding the listed metabolites	**Top quintile vs remaining quintiles**						
	Full model			24.77	4.58E-54	21.18	2.16E-26
	1,5-anhydroglucitol (1,5-AG)	-	-	19.98	4.59E-51	14.79	8.87E-24
	mannose	-	-	20.55	3.21E-51	16.90	1.37E-24
	glucose	-	-	23.34	3.60E-54	21.38	1.33E-26
	1,5-anhydroglucitol (1,5-AG)	mannose	-	20.18	5.88E-50	13.89	1.49E-22
	1,5-anhydroglucitol (1,5-AG)	glucose	-	18.39	6.49E-50	15.28	2.51E-24
	mannose	glucose	-	18.10	1.82E-50	13.19	5.72E-23
	1,5-anhydroglucitol (1,5-AG)	mannose	glucose	9.26	1.45E-35	5.96	3.33E-14
Excluding the listed metabolites	**Top decile vs remaining deciles**						
	Full model			31.87	2.74E-76	19.49	6.09E-37
	1,5-anhydroglucitol (1,5-AG)	-	-	24.40	6.15E-68	14.95	3.71E-32
	mannose	-	-	34.49	1.76E-79	19.10	1.04E-36
	glucose	-	-	28.48	1.47E-73	18.57	3.38E-36
	1,5-anhydroglucitol (1,5-AG)	mannose	-	23.47	2.46E-64	14.94	7.07E-31
	1,5-anhydroglucitol (1,5-AG)	glucose	-	21.03	1.11E-63	11.76	4.72E-28
	mannose	glucose	-	30.45	3.69E-75	15.85	4.59E-33
	1,5-anhydroglucitol (1,5-AG)	mannose	glucose	13.91	1.64E-49	7.40	3.21E-19

Table S4. Performance of MRS_{qcbio} in QBB using top quintiles and deciles.

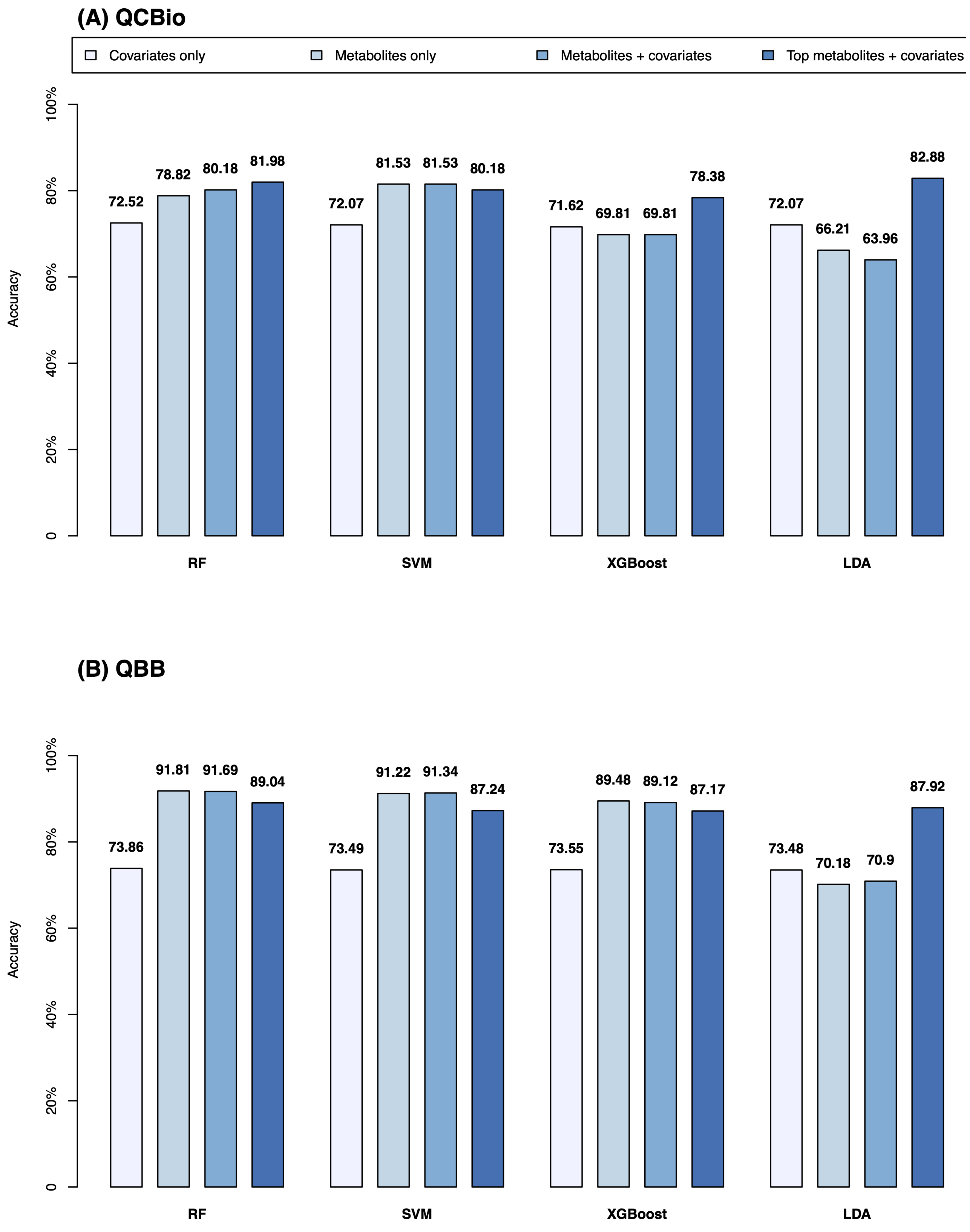

Figure S2. Accuracy of various ML models to predict T2D for 4 different settings. The ML models, RF, SVM, XGboost, and LDA are presented on the X-axis. Y-axis is the Accuracy. The numbers on top of each bar are the actual Accuracy values (in %). The bars from left to right of each model, colored as ice blue represent the Covariates only setting; light blue represent Metabolites only setting; cyan represent Metabolites and covariates setting; and dark blue represent Top 20 metabolites + covariates setting. (A) The Accuracy was computed in the QCBio cohort; and (B) The Accuracy was computed in the QBB cohort.

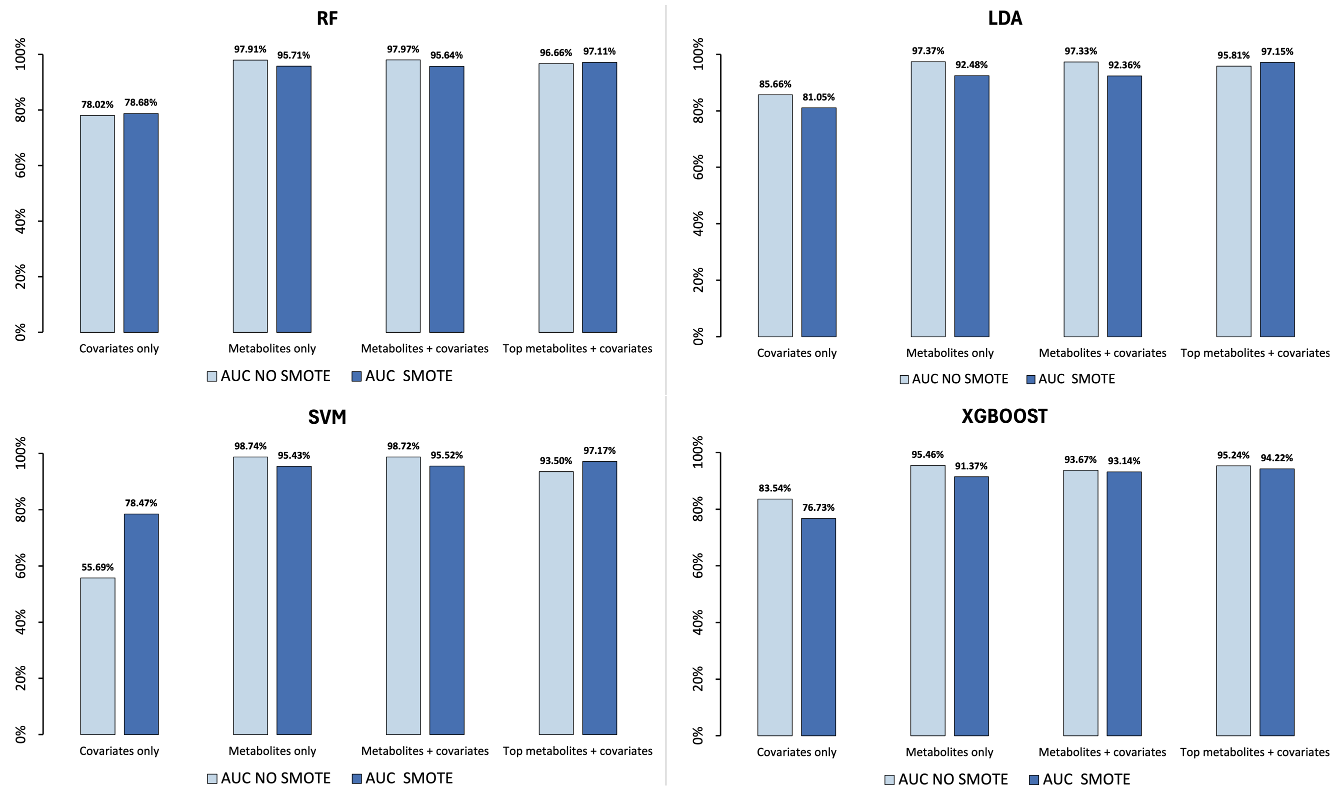

Figure S3. Area Under the Curve (AUC) comparison between SMOTE and no SMOTE results.

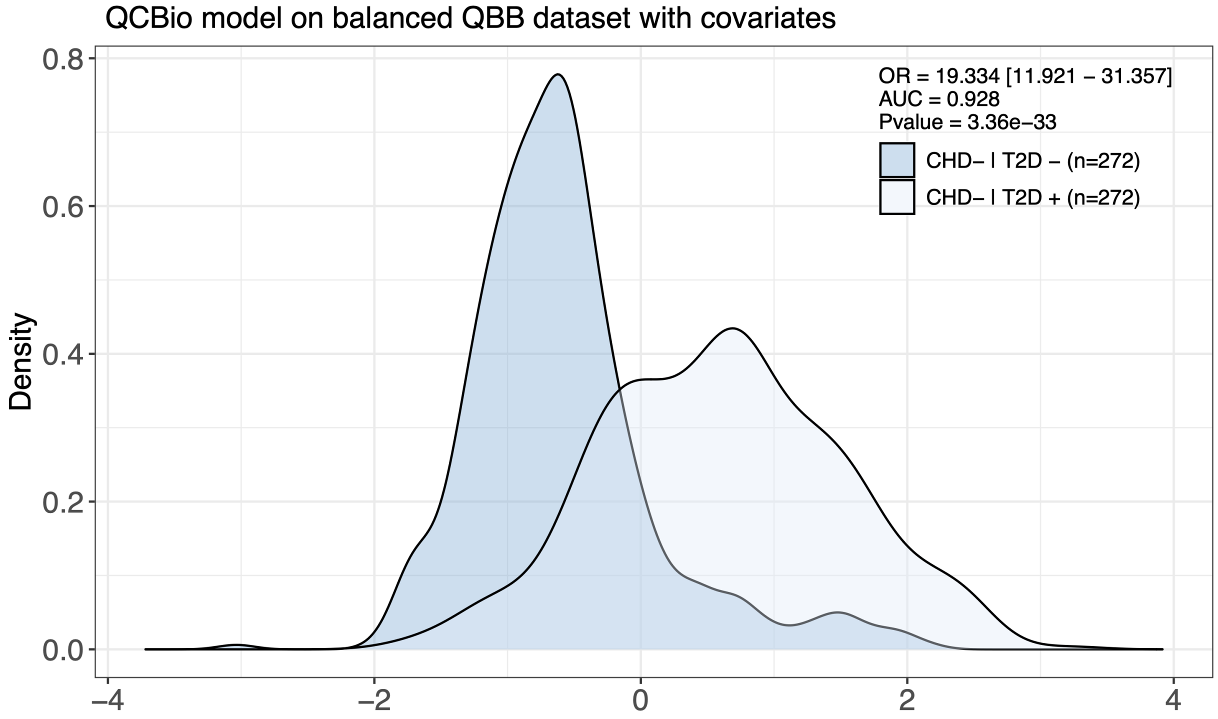

Figure S4. Distribution of the metabolite risk scores (MRS) for each of the reported classes. Model is trained in QCBio dataset and tested in a balanced QBB dataset. Covariates (Gender, BMI, Age) are included in the model. Light blue represents the T2D patients (CHD- | T2D+) and dark blue represents the healthy individuals (CHD- | T2D-). In top-right we report the OR value, AUC (0-1 scale), and the P value. The MRS was computed in the QCBio dataset and tested in the balanced QBB dataset.

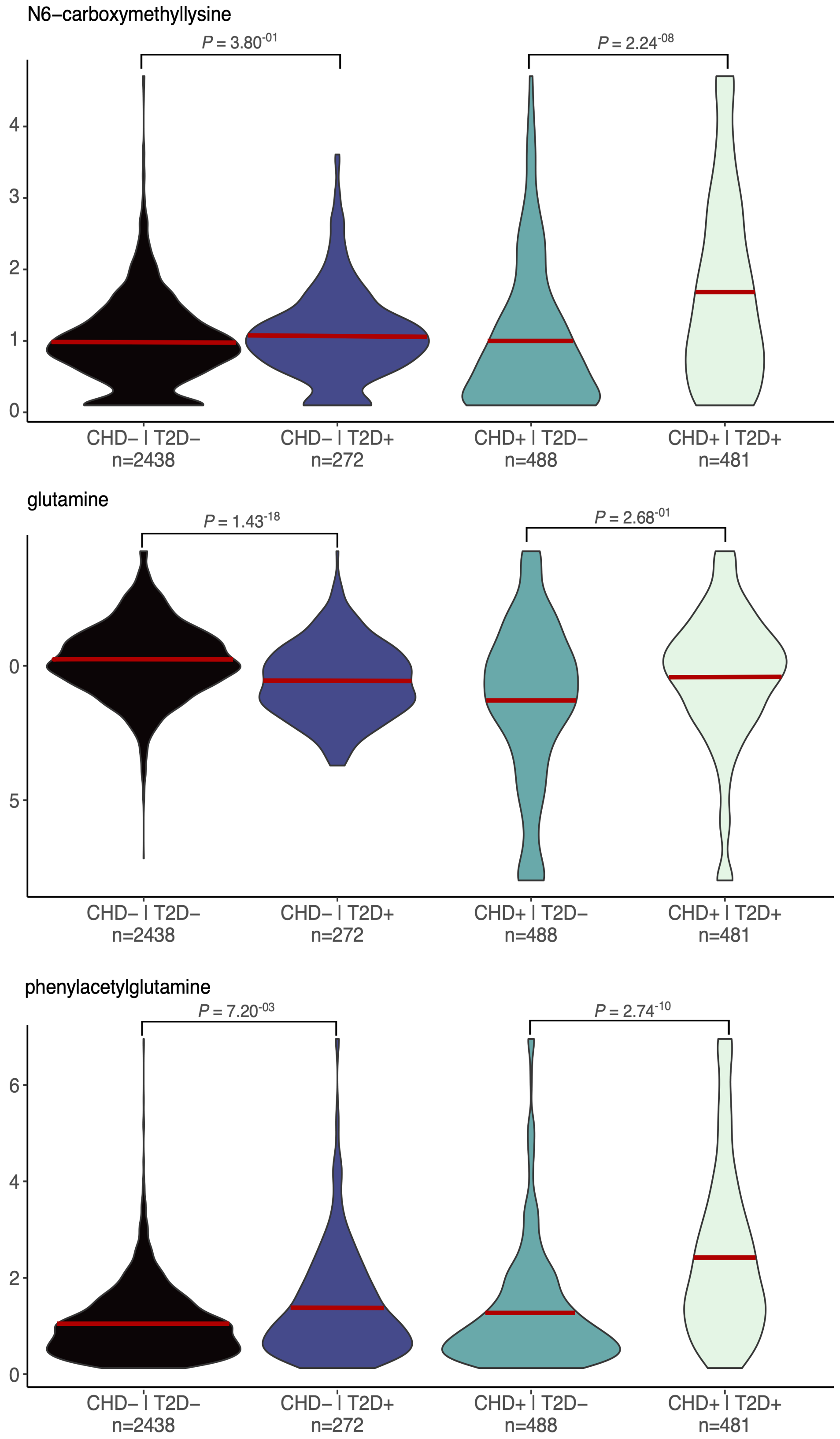

Figure S5. Statistics of the values of three metabolites. From left to right, black violins represent healthy patients (CHD- | T2D-), blue violins represent T2D patients (CHD- | T2D+), turquoise violins represent CHD patients (CHD+ | T2D-), and lime violins represent patients with both diseases (CHD+ | T2D+). The red line annotates the mean of the metabolite levels. (A) Statistics of N6-carboxymethyllysine values (B) Statistics of glutamine values (C) Statistics of phenylacetylglutamine values.
